# Comparative Analysis of Transcriptomes in Rhizophoraceae Provides Insights into the Origin and Adaptive Evolution of Mangrove Plants in Intertidal Environments

**DOI:** 10.3389/fpls.2017.00795

**Published:** 2017-05-16

**Authors:** Wuxia Guo, Haidan Wu, Zhang Zhang, Chao Yang, Ling Hu, Xianggang Shi, Shuguang Jian, Suhua Shi, Yelin Huang

**Affiliations:** ^1^State Key Laboratory of Biocontrol and Guangdong Provincial Key Laboratory of Plant Resources, School of Life Sciences, Sun Yat-sen UniversityGuangzhou, China; ^2^Department of Neurosurgery, First Affiliated Hospital of Sun Yat-sen UniversityGuangzhou, China; ^3^Chinese Academy of Sciences, South China Botanical GardenGuangzhou, China

**Keywords:** abiotic stress, extreme environment, adaptive evolution, mangrove, transcriptome, positive selection

## Abstract

Mangroves are woody plants that grow at the interface between land and sea in tropical and subtropical latitudes, where they exist in conditions of high salinity, extreme tides, strong winds, high temperatures, and muddy, anaerobic soils. Rhizophoraceae is a key mangrove family, with highly developed morphological and physiological adaptations to extreme conditions. It is an ideal system for the study of the origin and adaptive evolution of mangrove plants. In this study, we characterized and comprehensively compared the transcriptomes of four mangrove species, from all four mangrove genera, as well as their closest terrestrial relative in Rhizophoraceae, using RNA-Seq. We obtained 41,936–48,845 unigenes with N50 values of 982–1,185 bp and 61.42–69.48% annotated for the five species in Rhizophoraceae. Orthology annotations of Gene Ontology, Kyoto Encyclopedia of Genes and Genomes, and Clusters of Orthologous Groups revealed overall similarities in the transcriptome profiles among the five species, whereas enrichment analysis identified remarkable genomic characteristics that are conserved across the four mangrove species but differ from their terrestrial relative. Based on 1,816 identified orthologs, phylogeny analysis and divergence time estimation revealed a single origin for mangrove species in Rhizophoraceae, which diverged from the terrestrial lineage ~56.4 million years ago (Mya), suggesting that the transgression during the Paleocene–Eocene Thermal Maximum may have been responsible for the entry of the mangrove lineage of Rhizophoraceae into intertidal environments. Evidence showed that the ancestor of Rhizophoraceae may have experienced a whole genome duplication event ~74.6 Mya, which may have increased the adaptability and survival chances of Rhizophoraceae during and following the Cretaceous–Tertiary extinction. The analysis of positive selection identified 10 positively selected genes from the ancestor branch of Rhizophoraceae mangroves, which were mainly associated with stress response, embryo development, and regulation of gene expression. Positive selection of these genes may be crucial for increasing the capability of stress tolerance (i.e., defense against salt and oxidative stress) and development of adaptive traits (i.e., vivipary) of Rhizophoraceae mangroves, and thus plays an important role in their adaptation to the stressful intertidal environments.

## Introduction

Mangroves are a group of biogeographically and taxonomically diverse halophytes, predominantly trees that dominate tropical intertidal zones and estuaries (Tomlinson, 1986; Dassanayake et al., 2009). Mangrove habitats are extreme environments characterized by high salinity, flooding, hypoxia, wind, and high ultraviolet (UV) radiation in typically resource-poor conditions (Tomlinson, 1986; Cheeseman et al., 1991). As true extremophiles, mangroves have developed specific morphological and physiological characteristics during their evolutionary process of adaptation, such as exposed breathing roots, extensive support roots, buttress roots, salt-excreting leaves, and viviparous water-dispersed propagules (Duke, 1992; Bandaranayake, 2002; Kathiresan, 2011). Thus, mangroves are a valuable resource for understanding and exploiting plant adaptation to extreme environments (Dassanayake et al., 2009, 2010).

The genetic bases underlying these adaptive traits of mangroves, however, are still virtually unknown, probably due to limited genomic information and lack of comprehensive comparative analyses. For instance, responses to stresses are usually governed by multigenes in plants, which implies that the investigation of individual genes cannot provide enough information to unravel the molecular mechanisms of stress resistance (Flowers, 2004; Huang et al., 2012). Mangroves today are represented in at least 20 families (Duke et al., 1998). With a multitude of structural adaptations reflecting responses to common environmental constraints, the mangrove community exemplifies one of the stronger cases for convergent evolution in the plant kingdom (Tomlinson, 1986; Ellison et al., 1999). As evidenced by pollen fossils, mangroves probably evolved from terrestrial rather than marine plants (Srivastava and Binda, 1991). Studies based on fossils and phylogenetic analysis have suggested that the diverse mangrove genera are of independent origin in different geologic epochs (Shi et al., 2005; Ricklefs et al., 2006). Despite the various interpretations of the origin of mangroves, the consensus is that mangroves originated during the late Cretaceous near the Sea of Tethys (Plaziat et al., 2001; Saenger, 2002; Dassanayake et al., 2009). The exact phylogenetic position, divergence time, and species radiation within genera and families are still unclear and are of great interest to many botanists.

Over the past decade, comparative analyses of mangrove species using RNA sequencing have examined the roles of specific sequence divergences, gene components, and natural selection in the adaptation of mangroves at the genomic level, mostly based on the analysis of one representative mangrove species in different families (Yang et al., 2015a; Li et al., 2016). However, the plants used in these comparative studies are either distantly related (different family or class), or just one or two mangrove congeners, which makes it difficult to distinguish adaptive processes and identify convergent evolution from aspects caused by phylogenetic effects. Thus, comparative genomic studies involving mangrove taxa in higher taxonomic categories in a phylogenetic framework will help elucidate the adaptations of mangrove plants to stressful intertidal zones and facilitate studies in convergent evolution.

Another phenomenon that has long fascinated botanists is polyploidy, or whole-genome duplication (WGD), which has been widely recognized as an important mechanism of plant speciation and evolution (Doyle et al., 2008; Barker et al., 2009; Soltis et al., 2009). Despite the potential for ecological and genomic havoc, WGD may have facilitated evolutionary innovations and adaptations through the generation of novel genetic material, regulation of the following diploidization process, and regulation of the accompanying changes in gene expression and chromosome rearrangements (Barker et al., 2009; Vanneste et al., 2013). WGDs have been uncovered in many phylogenetic clades and are associated with the success of angiosperms (Ramsey and Schemske, 1998; De Bodt et al., 2005; Fawcett et al., 2009; Soltis et al., 2009; Vanneste et al., 2015). Studies of WGDs, however, have rarely been reported for mangroves (He et al., 2015). The analysis of genes under positive selection, a potential force of adaptive divergence that might have driven the evolution of mangroves and their divergence from terrestrial relatives, has also been limited in mangrove studies (He et al., 2015; Yang et al., 2015a). The identification of positively selected genes (PSGs) and WGDs of mangroves will provide more insight into their evolutionary success in the harsh habitats.

The Rhizophoraceae is a family of tropical and subtropical flowering plants comprising ~16–18 genera distributed mainly in the Old World (Hou, 1958; Van Vliet, 1976). Although it is often described as a mangrove family, only four genera (i.e., *Bruguiera, Kandelia, Rhizophora*, and *Ceriops*), including 16 species, live exclusively in mangrove habitats (Tobe and Raven, 1988). Rhizophoraceae mangroves are widely distributed along tropical coastlines and the terrestrial species grow in both primary and successional moist forests (Juncosa and Tomlinson, 1988). To cope with common environmental constraints, these mangrove taxa have developed similar physiological and morphological adaptive strategies that distinguish them from their terrestrial relatives, such as viviparous propagules and salt exclusion (Parida and Jha, 2010). These characteristics make them ideal systems for investigating the adaptive evolution of mangroves in extreme environments while minimizing phylogenetic influences and other background noises. Molecular phylogenetic analysis also supports the placement of these four mangrove genera in the tribe Rhizophoreae, with the tribe Gynotrocheae being their closest terrestrial relative (*Carallia* has a basal position in this tribe; Schwarzbach and Ricklefs, 2000). Interestingly, some non-mangrove members of the family, such as *Carallia brachiata*, also have aerial stilt roots, suggesting that this character preceded the entry of the Rhizophoraceae into coastal habitats (Dinghou, 1958). To the best of our knowledge, there have been no in-depth genomic-level comparative studies on the multiple genera of Rhizophoraceae. In this study, we performed a comprehensive transcriptome analysis of four mangrove species (*Bruguiera gymnorhiza, Kandelia obovata, Rhizophora apiculata*, and *Ceriops tagal*) and one terrestrial species (*Ca*. *brachiata*), based on RNA-Seq. We set up large-scale unigene datasets for these Rhizophoraceae taxa and explored possible mangrove-specific genetic components, reconstructed the phylogeny and dated the divergence between these mangrove genera and their terrestrial relatives, and identified WGDs and PSGs in these species and assessed their association with the adaptive evolution of mangroves in the harsh intertidal habitats. The characterization of the transcriptomes of these species may provide a useful complement to understanding the molecular mechanisms underlying the adaptation of mangroves to intertidal zones.

## Materials and methods

### Plant materials and RNA sequencing

*Bruguiera gymnorrhiza, Kandelia obovata*, and *Rhizophora apiculata* were collected from Dongzhai Harbor Nature Reserve, Hainan, China, and *C. brachiata* was collected from Baiyun Mount, Guangzhou, China. For each species, total RNA was separately isolated from fresh young leaves and roots of the same individual using a modified CTAB method (Fu et al., 2004) and quantified by NanoDrop (Thermo Fisher Scientific Inc., Waltham, MA, USA). The leaves and roots were rinsed thoroughly in running water to remove the dust and surface-sterilized with 75% ethanol solution before RNA extraction to avoid exogenous contamination. Then, equal amounts of total RNA from the leaves and roots of a species were uniformly mixed and delivered to the Beijing Genome Institute (Shenzhen, China) for cDNA library construction and sequencing; mRNAs were extracted using Oligotex™-dT30 (TaKaRa, Dalian, China) and fragmented ultrasonically. The fragmented mRNAs were converted into double-stranded cDNAs using random primers and then adaptors were ligated to both ends. The purified cDNA libraries were sequenced using the GAII platform (Illumina Inc., San Diego, CA, USA) with a read length of 90 bp and insertion size of 200 bp. For comparison, raw reads of *Ce. tagal* were obtained from Yang et al. (2015b) and processed as described above.

### *De novo* assembly and annotation

To ensure cleanness of our data, we examined potential exogenous contamination in both raw sequencing reads (before assembly) and assembled unigenes (see below), and appropriate filter would be applied accordingly if necessary. The sequences were first searched against the NCBI's non-redundant database, and then the levels of contamination were assessed by calculating the proportion of top blast hits in different taxa categories, including plant, animal, fungi, bacteria, archae, viruses and viroids, and other. For the 1,000 short reads randomly selected from each of the four libraries (*B. gymnorrhiza, K. obovata, R. apiculata*, and *C. brachiata*) sequenced in this study, most best hits resulted from “plant” (97.42–98.22%), a small amount of best hits resulted from “animal” (1.36–2.58%), “fungi” (0.00–0.85%), and “bacteria” (0.00–0.34%), and no best hits resulted from “archae,” “viruses and viroids,” and “other” (Supplementary Figure S1). Additionally, the levels of contamination were assessed for all assembled unigenes of each species using Blast search. Similar results were observed, in which most sequences (97.87–99.31%) received an annotation from plant (Supplementary Figure S2). These results indicated nearly negligible exogenous contamination in our samples.

To improve the overall quality of the sequencing reads, read pairs with average quality scores below 20 or with more than five ambiguous bases were filtered out using in-house perl scripts. The clean reads were *de novo* assembled into contigs using Trinity software (release 20110519; Grabherr et al., 2011) with the default parameters, and redundancies of the assembled contigs were removed using CAP3 (Huang and Madan, 1999) with an identity threshold of 99%. For each species, all usable reads were mapped to a non-redundant set of transcripts using MAQ (http://maq.sourceforge.net), and the average coverage for each sequence was calculated accordingly. Only sequences with an average coverage of not <1-fold were retained as reliable unigenes for further analysis. Reads per kilobase per million mapped reads (RPKM) for each unigene was calculated and used to quantify their relative abundance.

For functional annotation, the unigenes were initially searched against the NCBI non-redundancy (NR) protein database using BLASTX with an *e*-value cutoff of 10^−6^. Then the BLAST results were analyzed by Blast2GO (Conesa et al., 2005) and Gene Ontology (GO) terms were assigned to the annotated unigenes. For further avoiding potential influences of exogenous contamination on downstream analysis, GO-slim were applied in the last step of Blast2GO pipeline, and only annotations from plants were retained for GO enrichment analysis and other GO annotation-based comparative studies. The gene functions were further predicted and classified by searching against the Clusters of Orthologous Groups (COG) database. Kyoto Encyclopedia of Genes and Genomes (KEGG; Kanehisa and Goto, 2000) pathways were also assigned to the unigenes using the online KEGG Automatic Annotation Server. The distributions of GO terms at level 2 were plotted for three main categories: cellular component, biological process, and molecular function. Differences in gene numbers in a certain GO category (at all levels) between species were further tested for significance using a Fisher's exact test in GOBU (Lin et al., 2006). Significant differences in KEGG pathways and COG categories between species were also tested using Fisher's exact test.

### Identification of transcription factors and simple sequence repeats

The identification and classification of transcription factors (TFs) were carried out using iTAK software (version 1.2; http://bioinfo.bti.cornell.edu/cgi-bin/itak/index.cgi) based on the rules (required and forbidden pfam protein domains for each gene family) described in Perez-Rodriguez et al. (2010). To compare the TF classes between the terrestrial species and each mangrove species in Rhizophoraceae, Fisher's exact test was performed for each TF family in terms of its proportional representation among all TF genes.

### Phylogeny reconstruction and divergence time estimation

To clarify the phylogenetic relationships of the five Rhizophoraceae species, a phylogeny was constructed using transcriptome sequences of these species and the genomic sequences of *Populus trichocarpa* (as an outgroup). The open reading frame and protein sequences for unigenes of the five Rhizophoraceae species were obtained based on the BLASTX results. The coding sequences of *P*. *trichocarpa* genes and proteins were downloaded from Phytozome 9.1 (https://phytozome.jgi.doe.gov/pz/portal.html). Protein sequences of the six species were combined to perform all-against-all comparisons using BLASTP with an *E*-value cutoff of 10^−10^ and an identity threshold of 40%. The results were fed into the stand-alone OrthoMCL (Li et al., 2003) program using the default parameters, and putative orthologous groups were defined accordingly.

All derived single-copy orthologs were used to reconstruct the phylogenetic tree. Based on protein-sequence alignments generated in CLASTALW (v2.1; Larkin et al., 2007), the corresponding coding-sequence alignment for each orthologous group was obtained using Pal2nal (version 14; Suyama et al., 2006). For each species, all sites from the codon sequence alignments were concatenated to one supergene. A best substitution model was selected using the JModeltest2 program (Darriba et al., 2012), and PhyML (v3.0; Guindon et al., 2010) was used to reconstruct the phylogenetic tree using the best-fit model. Divergence time was estimated using the mcmctree program implemented in PAML 4.8 (Yang, 2007). In prior settings, the root constraint of the Rhizophoraceae species was set to 105–120 million years ago (Mya) (Davis et al., 2005; Xi et al., 2012), and the divergence time between *B*. *gymnorrhiza* and other mangrove species was set to 41.3–56 Mya, in accordance with the fossil record (Collinson, 1983; Graham, 2006).

### Identification and dating of WGD events

WGDs could be inferred from duplicate age distribution. For the identification of duplicate genes within a species, self-alignment was applied to the protein sequences of the species using BLASTP and then paralog groups were obtained using OrthoMCL (for methods and parameter settings, refer to the previous section). For each species, genes within a paralog group were identified as duplicates descended from a single gene. For all duplicate gene pairs, their divergence, in terms of substitutions per synonymous site per year (*K*_*S*_), was calculated using the maximum likelihood (ML) method in the codeml module of PAML. To remove duplication events that could bias the results, the dataset was further processed as follows: all duplicate pairs annotated with transposable elements were removed to reduce the effects of transposition-derived duplication, all duplicate pairs with *K*_S_ = 0 were removed to reduce identical genes missed by CAP3 assembly due to alternative splicing, simple hierarchical clustering was applied to reduce the multiplicative effects of multicopy gene families on *K*_*S*_-values, and all duplicate pairs with *K*_S_ > 2.0 were removed to avoid misleading of the mixture modeling caused by *K*_S_ saturation and stochasticity effects (Barker et al., 2008; Vanneste et al., 2015).

Peaks produced by ancient WGDs, or paleopolyploidy, are expected to be approximately Gaussian (Blanc and Wolfe, 2004). To identify the number of normal distributions and their positions that could produce the observed age distributions, EMMIX was utilized to fit a mixture model of normal distributions to the data using the ML method (McLachlan et al., 1999). SiZer (Chaudhuri and Marron, 1999) was also used for the further identification of significant features in the age distributions. To estimate the time that a putative WGD occurred, a peak value was obtained from the *K*_S_ distribution. Then, assuming a constant mutation rate, the WGD time could be estimated according to the formula *K*_S_ = 2T μ, where *K*_S_ is the peak value, T is the WGD time, and μ is the synonymous mutation rate of the species. After constructing an average *K*_S_ tree for the five Rhizophoraceae species and *P*. *trichocarpa* using all single-copy orthologs, the synonymous substitution rate of a species could be roughly measured based on the branch length and the previously defined divergence time. In this way, the average mutation rate was estimated to be 2.48 × 10^−9^ for *B*. *gymnorrhiza*. To better understand the retention pattern of WGD-derived duplicates, Fisher's exact test in GOBU was used to test for significant differences in gene numbers in each GO category between paleologs and non-paleologs of each species and between paleologs of mangrove and terrestrial species.

### Identification of candidate genes under positive selection

All identified single-copy orthologs were used to detect putative genes under positive selection in mangroves. The most recent common ancestor (MRCA) of the four mangrove species (i.e., *B*. *gymnorrhiza, K*. *obovata, R*. *apiculate*, and *Ce*. *Tagal*) was set as the foreground branch while the others were set as the background branch. The improved branch-site model and likelihood ratio test were adopted to test for positive selection using the codeml program implemented in the PAML package. To avoid false discoveries, the *P*-values were tested using the FDR method with a conservative 5% false discovery rate criterion.

## Results

### Transcriptome sequencing and assembly

Using the Illumina HiSeq™ 2000 platform, ~13 million pairs of raw reads with an average GC content of about 46.67% were obtained for each of four Rhizophoraceae species (i.e., *Bruguiera gymnorrhiza, Kandelia obovata, Rhizophora apiculata*, and *C. brachiate*; Supplementary Table S1). Transcriptome data from *Ce*. *tagal*, using the same sequencing technology from our previous work, were also reprocessed here for the benefit of better comparative study. Q20 percentages, a benchmark for assessing the performance of short-reads sequencing, were above 95% for all samples, indicating that the raw reads were of high quality. After removing adapter sequences and low-quality reads, a total of over 25 million 90 bp paired-end clean reads remained for subsequent analyses of each species.

Clean reads were *de novo* assembled using Trinity software, resulting in 55,434–67,678 contigs with average lengths of 715–859 bp and N50 values of 1,077–1,374 bp (Supplementary Table S2). To determine and further improve the quality of the assemblies, we removed redundancies in the assemblies and mapped the paired-end reads back to the generated contigs. In this way, 93.04–96.48% of the reads could be mapped back to the contigs, covering 95.27–97.22% of the reference sequences; the average mapping depth of the reference sequences ranged from 36.21× to 53.04×, supporting the accuracy of the assemblies and the saturation of sequencing depth for gene discovery (Supplementary Table S3). After removing unreliable transcripts with an average depth of <1×, 46,862, 48,845, 41,936, 44,875, and 47,788 unigenes were generated for *B*. *gymnorrhiza, K*. *obovata, R*. *apiculate, Ce*. *tagal*, and *Ca*. *brachiate*, respectively. Assembly details are provided in Supplementary Table S4.

Further analysis found that the length and GC% distribution patterns of all unigenes of the five species were similar to each other (Supplementary Figures S3A,B). The majority of the transcripts were between 200 and 1,000 bases (74.90–79.48% transcripts), followed by 1,000–2,000 bases (15.80–18.86% transcripts), and 2,000–3,000 bases (3.58–5.04%) (Supplementary Figure S3A). The N50 values and average lengths of the assembled transcripts were close among the four mangrove species (N50: 1,060–1,185 bp, average length: 706–763 bp) but relatively lower for the non-mangrove species *C*. *brachiata* (N50: 982 bp, average length: 695 bp). The average GC contents of the assembled sequences ranged from 43.08 to 43.85% in the five Rhizophoraceae species. To obtain a preliminary knowledge of the gene expression profile, the relative expression abundance of each unigene was estimated using the RPKM approach (Supplementary Table S5).

### Functional annotation and categorization

For the verification and annotation of the assembled unigenes, a homology search was initially conducted against the NCBI NR database for all unigenes using the BLASTX program, and the best-aligning results were selected to annotate the unigenes. Among the total sequences of the five species, 29,317–31,864 (61.42–69.86%) had significant hits in the NR database (Supplementary Tables S6, S7). Based on the BLAST similarity distributions, more than 28,000 (>96%) sequences of the five species exhibited an alignment identity >60% at the protein level, and the majority (>56%) of the best hits had *e*-values lower than 1e^−50^ (Supplementary Figures S4A,B). Organism distribution analysis based on BLASTX results showed that the two species that contained the most homologous genes among the five Rhizophoraceae species were *Ricinus communis* (38.55–40.64%) and *P. trichocarpa* (36.12–38.41%), belonging to Malpighiales, followed by *Vitis vinifera, Glycine max*, and *Arabidopsis thaliana* (for *B*. *gymnorrhiza* and *K*. *obovata*)/*Medicago truncatula* (for *R*. *apiculata, Ce*. *tagal*, and *Ca*. *brachiate*; Supplementary Figure S4C). Among the five species, only transcripts from *Ce*. *tagal* and *Ca*. *brachiata* (125 and 122, respectively) had homologous hits with *Lotus japonicus* genes.

Next, unigenes with homologs to previously annotated sequences in the NR database were annotated with GO terms using Blast2GO. In all, 27,086–28,865 unigenes (48.96–54.97%) were assigned to one or more GO terms (Supplementary Figure S4D) and classified into 36 different sub-categories (at level 2) belonging to the three main categories: cellular component (10), biological process (15), and molecular function (11). The results showed generally similar gene distribution patterns among the five Rhizophoraceae species in these categories (Supplementary Figure S5). Further, comparative analysis between each mangrove species and the non-mangrove species at all GO levels, however, identified 76 abundant sub-categories across all four comparisons (*P* < 0.05 after correction for multiple comparisons; Supplementary Figure S6; Supplementary Table S8). In the biological process category, most noticeably, candidate genes involved in responses to stimuli, signal transduction, gene expression, energy generation, and embryo development were highly represented in the four mangroves. In the cellular component category, genes related to peroxisomes, vacuoles, cytosol, and plasma membranes were significantly enriched in the four mangrove species. Within the molecular function category, GO terms corresponding to TF activity, protein binding, and lipid binding were abundant among unigenes of the four mangrove species.

In addition, all unigenes were subjected to a search against the COG database. In all, 12,210–12,812 unigenes could be assigned to COG classifications (Supplementary Figure S7; Supplementary Tables S7, S9). Among the 26 different functional classes, the “general function prediction only” was the largest group (3,141–3,387; 24.83–25.97%) in the five Rhizophoraceae species, followed by “transcription” (1,509–1,667; 12.19–12.69%), and “replication, recombination, and repair” (1,412–1,605; 11.49–12.07%). Compared to the non-mangrove species *Ca*. *brachiata*, genes related to “cytoskeleton” were overrepresented in *K*. *obovata* and genes involved in “RNA processing and modification” and “intracellular trafficking, secretion, and vesicular transport” were more abundant in the mangrove species *Ce*. *tagal* (*P* < 0.05).

### Functional classification of KEGG pathways

Annotation based on the KEGG pathway allows for an overview of active metabolic processes within an organism. To better understand their biological functions, we mapped all unigenes against the KEGG database. In total, 3,381–3,424 (6.82–7.95%) unigenes were grouped into 354–356 KEGG pathways from the five Rhizophoraceae species (Supplementary Tables S7, S10). These pathways were found among different categories, which included metabolism, cellular processes, genetic information processing, environmental information processing, and others, with “metabolism” having the highest share, 63.62–64.55% (2,112–2,199 transcripts), in all five species. The gene contents of each pathway were similar among the five Rhizophoraceae species, and no pathway (*P* > 0.05) was more enriched in unigenes of the four mangroves than in the non-mangrove species *Ca*. *brachiata*. The 12,646–18,845 (30.14–38.58%) unannotated unigenes after the NCBI NR, GO, COG, and KEGG orthology annotations may yet be uncharacterized genes or specific gene sets of these species.

### Transcription factors

Based on the consensus rules for requirements and forbiddance of protein domains of each TF gene family, a total of 1,132–1,337 putative TF genes from 55 TF families were identified for the five Rhizophoraceae species (Supplementary Table S11). Overall, TFs were relatively more abundant in the four mangrove transcriptomes (2.57–2.98%) than in the non-mangrove species *Ca*. *brachiata* (2.53%) (Supplementary Figure S8). Among all TFs identified, further analysis revealed shared overrepresentation of 11 TF gene families in the four mangrove species, with SRS genes significantly (*P* < 0.05) more enriched in *K*. *obovata* and MYB genes more enriched in *Ce*. *tagal* than in *Ca*. *brachiata*.

### Phylogeny, divergence time, and WGD

**Figure 1:**
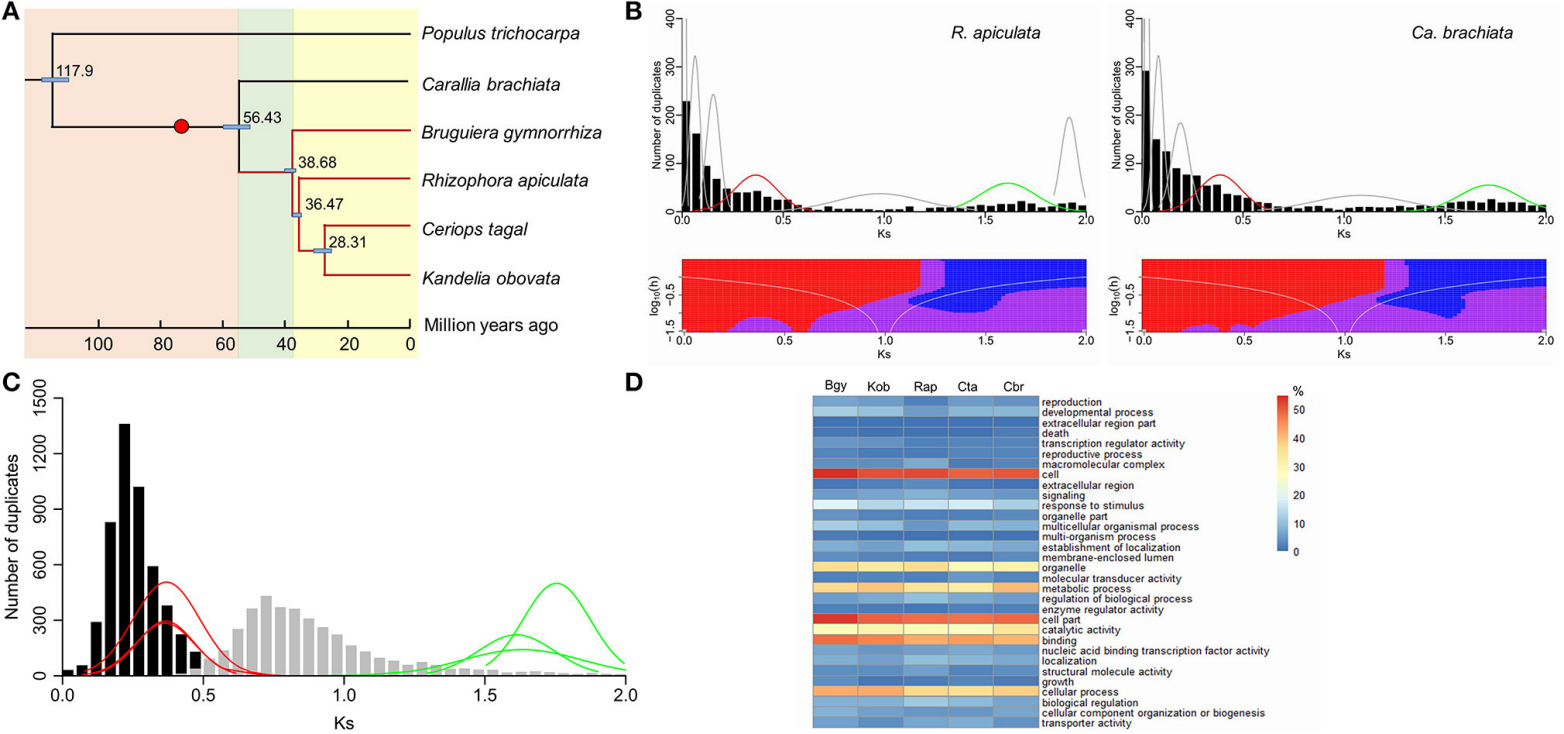
**Divergence time and whole-genome duplication in Rhizophoraceae. (A)** Phylogenetic relationships and divergence time of the five Rhizophoraceae species. **(B)** Histograms of gene duplication ages using the mixture model (above) and SiZer (below) analysis for the mangrove *R*. *apiculata* and terrestrial *Ca*. *brachiata*. **(C)** The relative placement of two inferred whole-genome duplications (WGDs) in Rhizophoraceae. **(D)** Gene Ontology (GO) annotations of paleologs from each Rhizophoraceae species examined. The red dot in the phylogenetic tree in **(A)** indicates the time when the inferred WGD shared by Rhizophoraceae occurred. Plots of normal distributions in **(B)** were fitted from mixture model analysis; red lines represent the younger WGDs shared in Rhizophoraceae, while green lines probably correspond to the older triplication shared by all angiosperms. Red and blue areas in SiZer maps indicate significantly decreasing and increasing slopes, respectively; purple represents no significant slope change; and gray indicates not enough data for the test. In **(C)**, histograms represent *K*_S_ distributions of ortholog genes between *Ca*. *brachiata* and *B*. *gymnorrhiza* (black), and between *Ca*. *brachiata* and *P*. *trichocarpa* (gray); red and green lines refer to **(B)**. In **(D)**, colors represent the percentage of paleologs that a particular GO category composes; Bgy, Kob, Rap, Cta, and Cbr represent *B*. *gymnorrhiza, K*. *obovata, R*. *apiculata, Ce*. *tagal*, and *Ca*. *brachiata*, respectively.

The existence of large datasets for both mangrove and non-mangrove species in Rhizophoraceae provides an opportunity to reexamine their phylogenetic relationships at the whole-genome scale, date their divergence, and more importantly, investigate the extent of genome duplication and its influence on the adaptation of mangroves to stressed intertidal environments. Using the transcriptome sequences of the five Rhizophoraceae species, 1,816 single-copy orthologous genes were identified and used for phylogeny reconstruction and estimation of divergence time. The results showed that in Rhizophoraceae, the earliest divergence was between mangrove and non-mangrove (*Carallia, Ca*. *brachiata*) species (~56.43 Mya, 95% CI: 52.5–61.1 Mya); in the mangrove taxa, *Bruguiera* (*B*. *gymnorrhiza*) diverged first 38.68 (95% CI: 37.6–41.0) Mya, followed by *Rhizophora* (*R*. *apiculata*) 36.47 (95% CI: 35.2–38.8) Mya, while the two sister genera *Ceriops* (*Ce*. *tagal*) and *Kandelia* (*K*. *obovata*) diverged ~28.31 (95% CI: 25.5–31.1) Mya (Figure 1A).

Further analyses of gene duplication using these transcriptome sequences provided clear evidence for at least two genome duplications in the history of Rhizophoraceae. A total of 149,318 unigenes for the five Rhizophoraceae species were clustered into 24,828 gene families. On average, each species was represented by 21,059 unigenes distributed across 17,467 gene families, with nearly 29.68% of the unigenes duplicated. *K*_S_-based age distributions were constructed for all duplicated gene pairs in the five transcriptomes (Figure 1B; Supplementary Figure S9). The histograms displayed two potential peaks in all species' age distributions, which most likely corresponded to two ancient polyploidies in Rhizophoraceae. The distribution corresponding to the younger peak in each species, however, was difficult to distinguish visually from the duplication-rich initial peaks (i.e., *K*_S_ = 0–0.1) because their tails overlap; the latter likely represent a mixture of tandem and other small-scale duplications (SSDs).

To separate the contribution of the log-transformed L-shaped SSD background exponential and WGD Gaussian functions from the overall age distribution, ML-mixture model analyses of the *K*_S_ distributions were employed. The five Rhizophoraceae taxa each demonstrated two normal distributions aligning with the two peaks observed in histograms: one with a peak center located at a *K*_S_ of 0.37, 0.37, 0.36, 0.37, and 0.39 and the other at a *K*_S_ of 1.75, 1.63, 1.61, 1.66, and 1.72 for *B*. *gymnorrhiza, K*. *obovata, R*. *apiculata, Ce*. *tagal*, and *Ca*. *brachiata*, respectively (Figure 1B; Supplementary Figure S9). Because several other smaller Gaussian functions were also identified by the mixture model, SiZer was used to further eliminate small stochastic deviations in the background SSD distribution. Not surprisingly, the younger peak mentioned above was not significant in SiZer maps of each species (Supplementary Figure S9), most likely because the prominent peak produced by the SSDs obscured the positive slope of the left tail of the peak corresponding to the WGD. This interpretation is supported by the significant declines near *K*_S_ = 0.3–0.4 in the SiZer maps of these species, which correspond to the right tails of the mixture model distributions. Altogether, duplicate gene analyses provided non-negligible evidence for two distributions centered at *K*_S_ = 0.30–0.43 and *K*_S_ = 1.38–1.72 as bona fide WGD signatures in Rhizophoraceae.

The use of 1,745 ortholog phylogenies yielded a mean divergence of *K*_S_ = 0.28 for *Ce*. *tagal* and other taxa in Rhizophoraceae and *K*_S_ = 0.89 for *Ce*. *tagal* and the outgroup *P*. *trichocarpa*. Taking into account the phylogenetic distribution of our sampled taxa and the proximity of their age distributions in scale and size (Figure 1C), the younger WGD events observed could be a shared paleopolyploidization near the base of Rhizophoraceae, while the older ones might correspond to a more ancient polyploidy shared by all eudicots. Based on the divergence time estimated above and the synonymous substitution rate calculated between taxa, the younger WGD could be roughly placed at ~74.57 (69.38–80.74) Mya, providing robust support for the preceding inference. GO analyses showed that the paleologs from this WGD were significantly different (*P* < 0.00001) from their non-paleologs for each species. Although no overrepresented GO categories in duplicates from the WGD were found to be shared among the four mangrove species in direct comparisons (Figure 1D), most of them had higher retention of genes related to responses to stimulus, TFs, or gene expression (Supplementary Table S12). Compared to *Ca*. *brachiata*, genes associated with protein binding, the cell periphery, plasma membranes, and biological regulation were overrepresented in paleologs of all sampled mangrove species except *Ce*. *tagal* (Supplementary Table S13).

### Genes under positive selection in rhizophoraceae mangrove species

**Figure 2:**
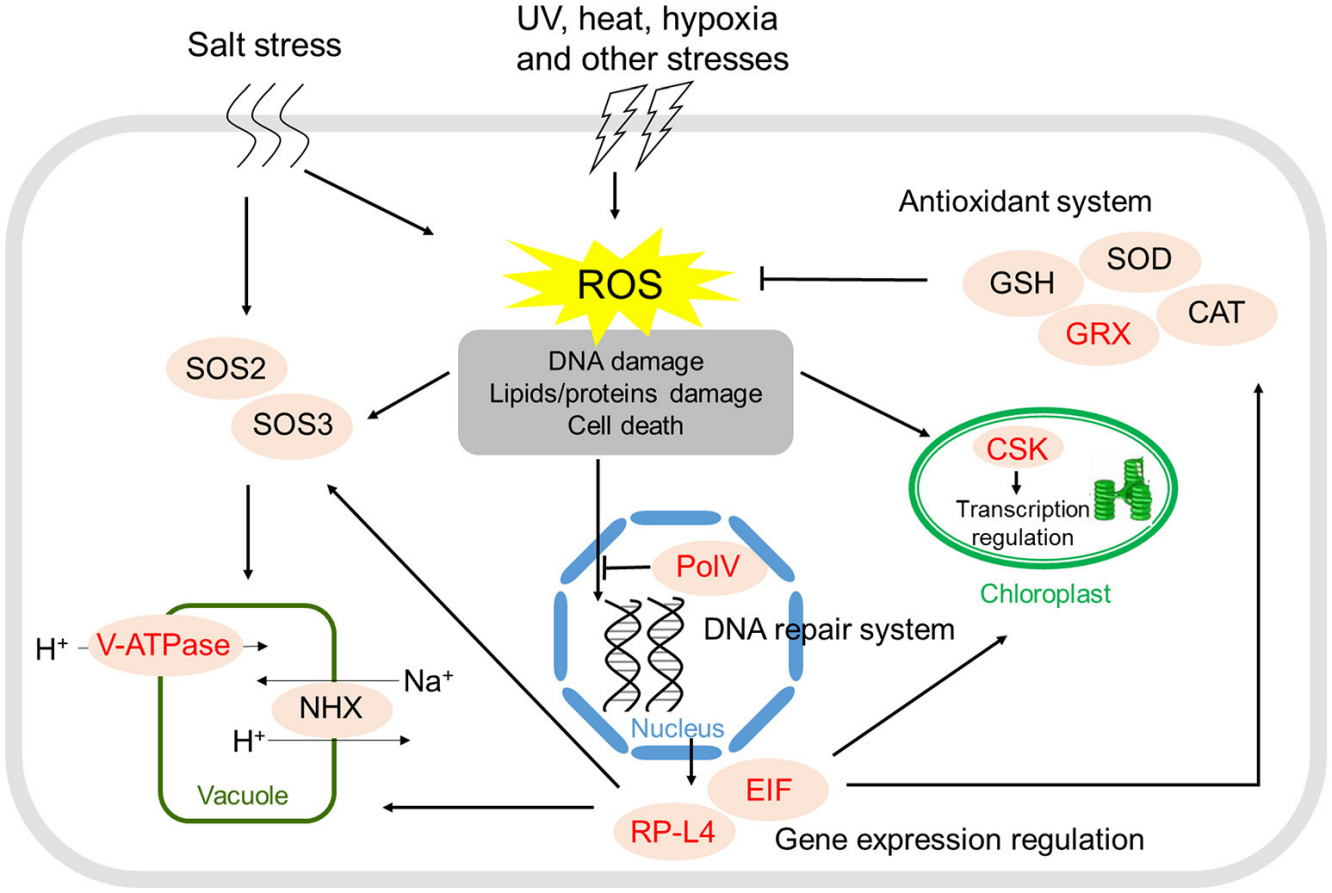
**Positively selected genes and stress resistance in the four Rhizophoraceae mangroves**. Positively selected genes are marked in red.

To identify candidate genes under positive selection along the branch of the MRCA of the four mangrove species in Rhizophoraceae, we further tested the 1,816 ortholog groups among them and the non-mangrove species *Ca*. *brachiata* using the improved branch-site likelihood method in the codeml module of PAML. A total of 10 genes were identified as PSGs in the MRCA of mangrove species in Rhizophoraceae, which were mainly related to stress responses, regulation of gene expression, and protein modification. Analyses of KEGG pathways showed that four PSGs were assigned to four different pathways (gf_9291 in ko03010, gf_9332 in ko03008, gf_9460 in ko03008, and gf_9846 in ko00520; Supplementary Table S14). Among which, two genes (gf_9291 and gf_9460) were both related to translation, one (gf_9332) was associated with energy metabolism, and the other (gf_9846) was associated with carbohydrate metabolism. Notably, gf_9332 encodes the subunit F of V-type H^+^-transporting ATPase (ATPeV1F), an important proton pump in plant cells that is of vital importance for plants' resistance to stress conditions and adaptation to the environment. Another important gene, gf_9611, encodes a redox enzyme glutaredoxin (GRX) acting in antioxidant defense, which can protect organisms from damage by reactive oxygen species (ROS) under various stresses (Figure 2).

## Discussion

### Refined genomic comparison identified mangrove-specific genetic components that might have benefited their adaptation to the harsh intertidal environments

In this study, we sequenced, assembled, and annotated the transcriptome of five species (four mangrove and one terrestrial species) in Rhizophoraceae using next-generation sequencing technology. A total of 41,936–48,845 unigenes were *de novo* assembled with mean lengths of 706–763 bp and GC contents of 43.08–43.85% for the five species, which displayed similar sequence characteristics (Supplementary Table S4; Supplementary Figure S3). The percentages of unigenes with NR annotations ranged from 61.42 to 69.48% among the five species. These results validated the quality of the assemblies and suggested wide coverage of gene models in the transcriptomes. The efficacy of such annotated transcripts was further verified by GO, COG, and KEGG pathway classification.

While mangroves have, evolutionarily, had a similar need to adapt to common environmental constraints, individual taxa representing at least 20 unrelated plant families (Duke et al., 1998) might have developed evolutionary convergence (i.e., in the sense of a mangrove “lifestyle”; Melzer et al., 2008). Whether there is genetic basis for this evolutionary convergence has long been an intriguing but controversial issue. The characteristics of mangrove transcriptomes can be evaluated by comparing phylogenetically diverse transcript populations that might reflect their evolutionary convergence. Dassanayake et al. (2009) compared the transcriptomes of two mangrove species, *Rhizophora mangle* and *Heritiera littoralis*, with two model terrestrial species, *A. thaliana* and *P. trichocarpa*. Their results strongly favored the interpretation that convergent evolution played a role at the transcriptome level in two diverse species that evolved separately to fit a common habitat, based on unusual similarities observed in the two mangrove transcriptome profiles. However, an absence of mangrove-specific genetic components has also been reported for other transcriptome-based studies on mangrove taxa (Chen et al., 2011; Yang et al., 2015a,b).

We compared the transcriptomes of four mangrove species and one terrestrial relative in Rhizophoraceae with respect to genes assigned in each GO class, KEGG pathway, and COG category. The highly qualified assemblies and consistent methodology used for each species ensured the reliability of the results. While the transcriptome profiles seemed to be analogous among all species in the larger GO categories (at level 2) (Supplementary Figure S5), further significant tests including all GO levels identified a total of 76 categories enriched and common across the four mangrove species compared to their terrestrial relative *Ca*. *brachiate*. Many of these categories have functions associated with responses to stimuli, peroxisomes, signal transduction, TF activity, reproduction and embryo development (Supplementary Figure S6; Supplementary Table S8). Compared with their terrestrial relatives, mangrove species are faced with major abiotic stresses of high salinity, periodic inundation, high temperature and ultraviolet radiation. Overrepresentation of stimuli response genes, especially those response to abiotic stimulus, could be very conductive to mangroves coping with these stress conditions. Besides, stress can result in excess of ROS in organisms. For mangroves that confront perennial severe abiotic stresses, ROS could be dramatically accumulated and thus cause serious damage to molecular components and structure of cells if it is not removed in time. Therefore, significant enrichment of genes related to ROS scavenging, i.e., peroxisomes, could be of great importance to effectively eliminate excess ROS and protect mangrove cells from oxidative and superoxide stress. Since mangroves suffer from periodic inundation and previous study in mangrove plant *Acanthus ilicifolius* showed that long-term inundation delays seed germination and deceases germination rate (Zhang et al., 2011), the reproduction related genes enriched in mangroves may be essential to improve their reproductive success in the flooding condition. Among which, genes involved in embryo development process could further be responsible for the atypical embryonic development leading to vivipary of mangroves, one of the most typical adaptive traits that support their survival in the intertidal zones. Collectively, these concurrently overrepresented categories may represent mangrove-specific genomic characteristics that reflect their evolutionary convergence, and the genes involved may be valuable candidates for further investigation of the global regulation networks of stress resistance at the transcriptome level in mangroves.

Our results, however, are not completely consistent with any previous report. Besides species specificity and differences in the tissue types used, the discrepancies between our study and previous ones may be due to differences in the completeness of datasets and the processing methods used. In Yang et al. (2015a), for instance, the comparison was only performed on larger GO categories (at level 2) rather than on more specific functional classes (i.e., at deeper levels); this could lead to the oversight of subtle but significant mangrove-specific genomic traits that might reflect the common pattern of adaptive evolution in mangroves. As another example, in Dassanayake et al. (2009), sequences that did not share a functional similarity with *Arabidopsis* were excluded from GO functional assignments even though they were similar to other plants. This probably resulted in an underestimate of the real number of genes assigned and a misidentification of categories of mangroves that are underrepresented compared to well-annotated model species.

### An ancient WGD in rhizophoraceae could have contributed to their divergence and adaptive evolution

The monophyly of the tribe Rhizophoreae, which comprises all four mangrove genera in Rhizophoraceae, was highly supported by our transcriptome data. In fact, among all mangrove families in which more than one mangrove genus is included, Rhizophoraceae is the only family in which all mangrove genera originated once (Shi et al., 2005). The relationships within Rhizophoraceae, particularly the isolated position of *Bruguiera* from other Rhizophoreae, is also evident in the phylogenetic tree, in agreement with previous morphological cladistic analyses and molecular phylogenetic reconstructions (Schwarzbach and Ricklefs, 2000). In *Bruguiera*, the seedling disperses initially with the fruit (Tomlinson, 1986), whereas in the more derived genera *Rhizophora, Kandelia*, and *Ceriops*, only the seedling disperses (Juncosa, 1982, 1984).

Polyploidization, or WGD, has been thought to increase organisms' tolerance to different environmental conditions due to advantages such as altered gene expression and increased genetic materials for selection (Fawcett et al., 2009). Besides the ancient triplication that all angiosperms most likely underwent (De Bodt et al., 2005; Soltis et al., 2009; Jiao et al., 2011), recent studies have demonstrated an additional more recent genome duplication that occurred in many different plant lineages within a same small time frame (60–70 Mya), associated with the Cretaceous–Tertiary (KT) boundary (~65 Mya) and proposed to have contributed to the survival and propagation of several plant lineages during or following the KT extinction event (Fawcett et al., 2009; Vanneste et al., 2014). Our research found evidence of two past genome duplications of the ancestor of Rhizophoraceae species (Figure 1; Supplementary Figure S9). The older genome duplication event, with peak *K*_S_ > 1, probably corresponds to the triplication shared with angiosperms and was not included in further WGD dating analysis, considering the saturation and stochasticity effects that might largely bias the results (Vanneste et al., 2013, 2015).

Based on the age distributions and phylogenetic relationships revealed by the transcriptome data, the younger genome duplication event was dated to ~74.6 Mya, which is close to the KT boundary and precedes the divergence of Rhizophoraceae mangrove and terrestrial taxa. As evidenced by fossil records and divergence time estimation (Figure 1A), the split between mangrove and terrestrial species in Rhizophoraceae occurred ~56.4 Mya, correlated with the extreme global warming event Paleocene–Eocene Thermal Maximum (PETM; ~55.5 Mya), which led to a large-scale rise in sea level (Handley et al., 2011). After this, Rhizophoraceae mangroves probably diversified in a relatively short time frame of ~10 Mya. Although we cannot establish a causal relationship between genome duplication and diversification in Rhizophoraceae taxa simply by the order of their occurrence, a reasonable hypothesis would be that the ancestor of Rhizophoraceae experienced a genome duplication around the KT boundary, which increased adaptability and chances of survival during the KT extinction; with a doubled set of genes and alleles available for selection, ancestral Rhizophoraceae populations underwent different evolutionary processes during the following PETM period as they recolonized different habitats (i.e., land and seashore), thus leading to the divergence of mangrove and terrestrial species; later differential adaptation to diverse living conditions within intertidal zones might have been responsible for the rapid radiation of Rhizophoraceae mangroves.

A process that begins immediately after a genome duplication is diploidization, a suite of molecular mechanisms that could lead to gene fractionation (return of many genes to a single copy) and chromosome changes, such as gene loss, mutation, and chromosomal rearrangements (Barker et al., 2008, 2009). While many genes return to a single copy by fractionation, some gene duplicates are preferentially maintained. This special class of paralogous genes, or paleologs, might reflect the genomic components or biological processes that are critical for a species' lifestyle. Generally, the genes retained in duplicate (derived from the genome duplication) had similar functional distributions among the five Rhizophoraceae species we examined (Figure 1D). But when compared with those returned to single copy (non-paleologs), the functional assignments of genes preferentially retained in duplicate (paleologs) were not completely consistent among species (Supplementary Table S12). A notable difference was that some paleologs were preferentially retained for genes associated with responses to stimuli, regulation of gene expression and/or embryo development in mangroves while not in the terrestrial *Ca*. *brachiata*. This finding matched the consistent enrichment of genes with such functions in the four Rhizophoraceae transcriptomes as mentioned above, and was also recovered when comparing only paleologs between *Ca*. *brachiata* and each mangrove species except *Ce*. *tagal* (Supplementary Table S13), hinting again the significance of these genes for mangroves' survival in intertidal environments. Therefore, it's reasonable to infer that the genome of the Rhizophoraceae ancestor doubled ~75 Mya, providing additional genetic materials for selection and thus increasing its survival chance during the KT extinction 65 Mya; while the global environment was dramatically changed by PETM 55.5 Mya, Rhizophoraceae populations recolonized different (i.e., coastal and inland) habitats, and the following preferential retention of duplicated genes related to stress response and reproduction/embryo development in coastal populations contributed to their adaptation to the intertidal environments and probably to their further divergence with inland populations. It's intriguing that no functional biases were observed between *Ca*. *brachiata* and *Ce*. *tagal* paleologs. Since *Ce. tagal* was in-house cultivated (Yang et al., 2015b) while others were directly sampled from their natural habitats, this observation could either represent virtual genomic features of the two species or be a consequence of distinct strategies and should be regarded with caution.

Previous studies have reported a pattern of parallel paleolog retention in several different lineages (Aury et al., 2006; Schranz and Mitchell-Olds, 2006; Scannell et al., 2007; Barker et al., 2008). Among these, the functions of genes preferentially retained in duplicate are substantially distinct between different lineages, therefore, the gene retention patterns of WGD-derived duplicates are consistent within a certain taxonomic category but vary among higher taxonomic categories (Barker et al., 2008). For instance, Compositae paleologs have been found to be significantly enriched for genes associated with structural components or cellular organization (Barker et al., 2008); paleologs in *Arabidopsis* and *Cleome* are enriched for genes associated with transcription and signaling (Schranz and Mitchell-Olds, 2006). For Rhizophoraceae as revealed by this study, although functional categories of paleologs were analogous among the five species examined (Figure 1D), none of them were significantly enriched across all species, mainly due to the small number of functional categories of preferentially retained paleologs in *K. obovata* (Supplementary Table S12). In contrast, we did detect a meager signature of gene retention that is consistent among mangroves and absent in the terrestrial species, i.e., the preferential retention of paleologs involved in stress response and/or embryo development. Because the split of Rhizophoraceae mangrove and terrestrial species (with a synonymous site divergence of 0.28 between terrestrial *Carallia* and the first mangrove genus to diverge, *Bruguiera*) was more recent than Compositae tribes (with an earliest tribal divergence of *K*_S_ = 0.62), the similarities in the duplicated gene retention patterns of Rhizophoraceae mangroves not only represent an indication of parallel paleolog retention resulting from phylogenetic effects, but also likely reflect mangrove-specific evolutionary processes for their adaptation to extreme living conditions.

### Positive selection of genes involved in stress response and embryo development were also important for mangroves' adaptation

Natural selection may be the major force that drives species evolution and divergence. Because mangrove and terrestrial plants differ markedly in their habitats with respect to various stresses, natural selection under different environments is likely to have driven their divergence and to have played an important role in mangroves' special adaptations. In the intertidal zones, high salinity tolerance, vivipary, and effective elimination of oxidative damage are three main adaptive abilities for mangroves. After multiple testing to avoid false positives, we identified 10 PSGs in the ancestor branch of Rhizophoraceae mangroves (Supplementary Table S14), most (8 of 10, 80%) of which were with known functions and were probably related to these adaptive traits. Among these eight PSGs, one gene (gf_9460) encodes the eukaryotic translation initiation factor 6 (eIF6), which is associated with the regulation of protein translation and is also critical in embryogenesis (Kato et al., 2010; Guo et al., 2011). Kato et al. (2010) found that the disruption of an eIF6 gene causes embryo lethal in *Arabidopsis*, demonstrating the critical role for eIF6 in embryogenesis. The sign of positive selection in eIF6 gene from Rhizophoraceae mangroves suggested this gene might be responsible for the atypical embryonic development leading to vivipary, an ability of a plant embryo to germinate while still attached to the parent which represents the most prominent phenotype that is well-developed in mangroves and extremely rare in non-mangroves (Tomlinson, 1986).

High salt stress in intertidal zones can lead to disruption of homeostasis in water potential and ion distribution of plants growth under such condition. It has been well-established that efficient vacuolar Na^+^ accumulation is an important step in maintaining ion homeostasis inside the cell in mangroves (Parida and Jha, 2010). This process is driven by the proton-motive force generated by V-type H^+^-ATPase, and increased expression of such proteins can improve salt tolerance (Baisakh et al., 2012). A gene (gf_9332) encoding subunit F of Vacuolar-type H^+^-ATPase (ATPeV1F) showed signal of positive selection and was generally upregulated in the four mangroves examined (Figure 2; Supplementary Table S14), suggesting that improvement of Na^+^ accumulation efficiency through positive selection and expression alteration of this gene could probably be one of the main strategies for Rhizophoraceae mangroves to increase their salt tolerance capability.

For mangroves in stressful intertidal habitats, environmental stress (e.g., UV or heat exposure) can lead to dramatic increases in ROS levels and result in significant cellular damage, such as damage to DNA, RNA, and proteins (Kipp and Boyle, 2013). Among the eight PSGs, one gene (gf_9611) encodes a small ubiquitous glutathione-dependent oxidoreductase glutaredoxin (GRX), which plays a crucial role in the response to oxidative stress (Figure 2). GRX-mediated regulation of cellular redox homeostasis may play a crucial role in posttranslational modifications of target proteins involved in organ development and defense responses against biotic and abiotic stresses in plants (Li, 2014). Besides, gene gf_7405 encodes a Y-family DNA polymerase DNA Polymerase V (PolV), which is capable of performing DNA translation synthesis (Yang, 2003) and is only expressed in cells during the SOS response (Fuchs and Fujii, 2013). PolV is very tightly regulated at different expression levels and under different mechanisms to avoid its activity unless absolutely necessary for the cell (Fuchs and Fujii, 2013). Positive selection of these two genes might collectively plays an important role for the effective scavenging and repairing of damage in Rhizophoraceae mangroves caused by oxidative and superoxide stresses under high salinity, high temperature and UV radiation conditions, thus improve their adaptive capability in intertidal zones.

## Conclusion

In summary, we sequenced, assembled, and characterized the transcriptomes of four mangrove species and a terrestrial relative in Rhizophoraceae. While the transcriptome profiles of the five Rhizophoraceae species were generally conserved with respect to GO, KEGG pathway, and COG assignments, enrichment tests also identified remarkable functional categories that are commonly overrepresented across the four mangrove species but differ from their terrestrial relative, probably reflecting mangrove-specific gene components that are essential for their adaptation. Phylogenetic analyses and divergence time estimations indicated that the initial entry of the Rhizophoraceae mangroves into the intertidal zones might have occurred around the PETM period, suggesting a potential causal association between the derived transgression and the origin of mangroves in Rhizophoraceae. Further analysis indicated a genome duplication event shared by Rhizophoraceae species near the KT boundary, which at least partly contributed to their divergence and adaptive evolution. Positive selection also played an important role in the process of adaptation to harsh intertidal zones. Our comprehensive transcriptome analysis has not only enriched the genomic sequence resources for Rhizophoraceae species but also largely increases our understanding of the origin and evolutionary adaptations of Rhizophoraceae mangroves in stressful intertidal environments.

## Deposited data

The RNA-seq datasets generated by using Illumina-Solexa platform are available from the NCBI Sequence Read Archive database (SRA; http://www.ncbi.nlm.nih.gov/sra) under experiment number accession SRX2342631, SRX2347535, SRX2347542, and SRX2347543.

## Author contributions

YH designed the study. XS, SS, and SJ collected materials. WG, HW, and LH performed experiments. WG, LH, ZZ, and CY analyzed and interpreted the data. WG, LH, and YH wrote the manuscript. All authors read and approved the final manuscript.

### Conflict of interest statement

The authors declare that the research was conducted in the absence of any commercial or financial relationships that could be construed as a potential conflict of interest.
