# Supplementary material for: Comparative Analysis of Transcriptomes in Rhizophoraceae Provides Insights into the Origin and Adaptive Evolution of Mangrove Plants in Intertidal Environments
Source: Front Plant Sci. 2017 May 16;8:795. doi: 10.3389/fpls.2017.00795 (PMC5432612; doi:10.3389/fpls.2017.00795)
Supplement: Supplementary file 1 [file SupplementaryFigures1-9andTables1-6.ZIP › Supplementary_Table_S1.docx]

**Supplementary Table S1 | Raw data from RNA-seq of the four species sequenced in this study.**

| Sample name | Raw reads | Raw reads length (bp) | Reads length (nt) | ≥Q20 percentage (%) | N percentage (%) | GC percentage (%) |
| --- | --- | --- | --- | --- | --- | --- |
| *K. obovata* | 12,949,109 | 2,330,839,620 | 90 | 98.21 | 0 | 45.48 |
| *B. gymnorrhiza* | 13,677,779 | 2,462,000,220 | 90 | 95.95 | 0.02 | 46.93 |
| *R. apiculata* | 12,723,393 | 2,326,707,720 | 90 | 96.63 | 0 | 47.59 |
| *Ca. brachiata* | 13,655,854 | 2,458,053,720 | 90 | 95.32 | 0 | 46.67 |

Q20 percentage shows the proportion of the sequenced bases with quality score ≥20.
