# Supplementary material for: Comparative Analysis of Transcriptomes in Rhizophoraceae Provides Insights into the Origin and Adaptive Evolution of Mangrove Plants in Intertidal Environments
Source: Front Plant Sci. 2017 May 16;8:795. doi: 10.3389/fpls.2017.00795 (PMC5432612; doi:10.3389/fpls.2017.00795)
Supplement: Supplementary file 1 [file SupplementaryFigures1-9andTables1-6.ZIP › Supplementary_Table_S2.docx]

**Supplementary Table S2 | Summary of raw contigs assembled by Trinity.**

| Name | n:200 | n:N50 | Min | Median | Mean | N50 | Max | Total number of bases |
| --- | --- | --- | --- | --- | --- | --- | --- | --- |
| *B. gymnorrhiza* | 67,678 | 13,291 | 200 | 535 | 859 | 1,374 | 10,275 | 58.14e6 |
| *K. obovata* | 65,844 | 12,221 | 200 | 496 | 838 | 1,364 | 11,199 | 55.18e6 |
| *R. apiculata* | 55,434 | 11,131 | 200 | 541 | 858 | 1,370 | 1,089 | 47.61e6 |
| *Ce. tagal* | 58,562 | 11,308 | 201 | 497 | 815 | 1,317 | 8,959 | 47.76e6 |
| *Ca. brachiata* | 60,287 | 12,067 | 201 | 446 | 715 | 1,077 | 7,629 | 43.13e6 |
