# Supplementary material for: Comparative Analysis of Transcriptomes in Rhizophoraceae Provides Insights into the Origin and Adaptive Evolution of Mangrove Plants in Intertidal Environments
Source: Front Plant Sci. 2017 May 16;8:795. doi: 10.3389/fpls.2017.00795 (PMC5432612; doi:10.3389/fpls.2017.00795)
Supplement: Supplementary file 1 [file SupplementaryFigures1-9andTables1-6.ZIP › Supplementary_Table_S3.docx]

**Supplementary Table S3 | Mapping statistics of the five Rhizophoraceae species.**

|  | *B. gymnorrhiza* | *K. obovata* | *R. apiculata* | *Ce. tagal* | *Ca. brachiata* |
| --- | --- | --- | --- | --- | --- |
| Total number of reads | 26,606,012 | 25,898,218 | 25,446,786 | 47,612,972 | 27,243,726 |
| Mapped reads | 25,668,954 | 24,918,004 | 24,445,266 | 45,566,128 | 25,346,268 |
| Reference length (bp) | 48,732,742 | 47,650,919 | 42,787,770 | 42,880,291 | 39,846,365 |
| Mapped length (bp) | 46,426,394 | 46,232,857 | 41,599,964 | 41,476,151 | 38,896,542 |
| Mapped percent (%) | 95.27 | 97.02 | 97.22 | 96.73 | 97.62 |
| Average depth (×) | 41.83 | 43.14 | 47.45 | 36.21 | 53.04 |
| Total number of contigs | 58,143 | 58,639 | 50,556 | 53,261 | 56,189 |
| # of contigs with depth ≥1 | 46,862 | 48,845 | 41,963 | 44,875 | 47,788 |
| % of contigs with depth ≥1 | 80.60 | 83.30 | 83.00 | 84.25 | 85.05 |
