# Supplementary material for: Comparative Analysis of Transcriptomes in Rhizophoraceae Provides Insights into the Origin and Adaptive Evolution of Mangrove Plants in Intertidal Environments
Source: Front Plant Sci. 2017 May 16;8:795. doi: 10.3389/fpls.2017.00795 (PMC5432612; doi:10.3389/fpls.2017.00795)
Supplement: Supplementary file 1 [file SupplementaryFigures1-9andTables1-6.ZIP › Supplementary_Table_S4.docx]

**Supplementary Table S4 | Summary of final assemblies of the five Rhizophoraceae species.**

|  | *B. gymnorrhiza* | *K. obovata* | *R. apiculata* | *Ce. tagal* | *Ca. brachiata* |
| --- | --- | --- | --- | --- | --- |
| Number of unigenes | 46,862 | 48,845 | 41,963 | 44,875 | 47,788 |
| Average length (bp) | 729 | 706 | 763 | 757 | 695 |
| Median length (bp) | 459 | 432 | 482 | 458 | 450 |
| Min length (bp) | 200 | 200 | 200 | 200 | 200 |
| Max length (bp) | 10,275 | 11,199 | 10,893 | 8,959 | 7,629 |
| N50 (bp) | 1,081 | 1,060 | 1,170 | 1,185 | 982 |
| Nn50 | 9,536 | 9,545 | 8,574 | 8,670 | 10,030 |
| Total length (bp) | 34,141,102 | 34,491,405 | 32,009,260 | 33,981,949 | 33,216,842 |
