# Supplementary material for: Comparative Analysis of Transcriptomes in Rhizophoraceae Provides Insights into the Origin and Adaptive Evolution of Mangrove Plants in Intertidal Environments
Source: Front Plant Sci. 2017 May 16;8:795. doi: 10.3389/fpls.2017.00795 (PMC5432612; doi:10.3389/fpls.2017.00795)
Supplement: Supplementary file 1 [file SupplementaryFigures1-9andTables1-6.ZIP › Supplementary_Table_S6.docx]

**Supplementary Table S6 | Summary of the annotation statistics based on NCBI NR, GO, COG, and KEGG orthology.**

|  | *B. gymnorrhiza* | *K. obovata* | *R. apiculata* | *Ce. tagal* | *Ca. brachiata* |
| --- | --- | --- | --- | --- | --- |
| Total number of unigenes | 46,862 | 48,845 | 41,963 | 44,875 | 47,788 |
| #Unigenes with hits to NCBI NR database | 30,369 | 30,000 | 29,316 | 29,700 | 31,863 |
| #Unigenes assigned for GO terms | 23,979 | 23,916 | 23,068 | 23,154 | 24,882 |
| #Unigenes with COG annotation | 12,812 | 12,589 | 12,284 | 12,210 | 13,639 |
| #Unigenes assigned to KEGG pathways | 3,381 | 3,334 | 3,330 | 3,314 | 3,424 |
| Total annotated unigenes | 30,870 (65.87%) | 30,493 (62.43%) | 29,710 (70.80%) | 29,982 (66.81%) | 32,430 (67.86%) |
