# Supplementary figures and images for: Comparative Analysis of Transcriptomes in Rhizophoraceae Provides Insights into the Origin and Adaptive Evolution of Mangrove Plants in Intertidal Environments
Source: Front Plant Sci. 2017 May 16;8:795. doi: 10.3389/fpls.2017.00795 (PMC5432612; doi:10.3389/fpls.2017.00795)

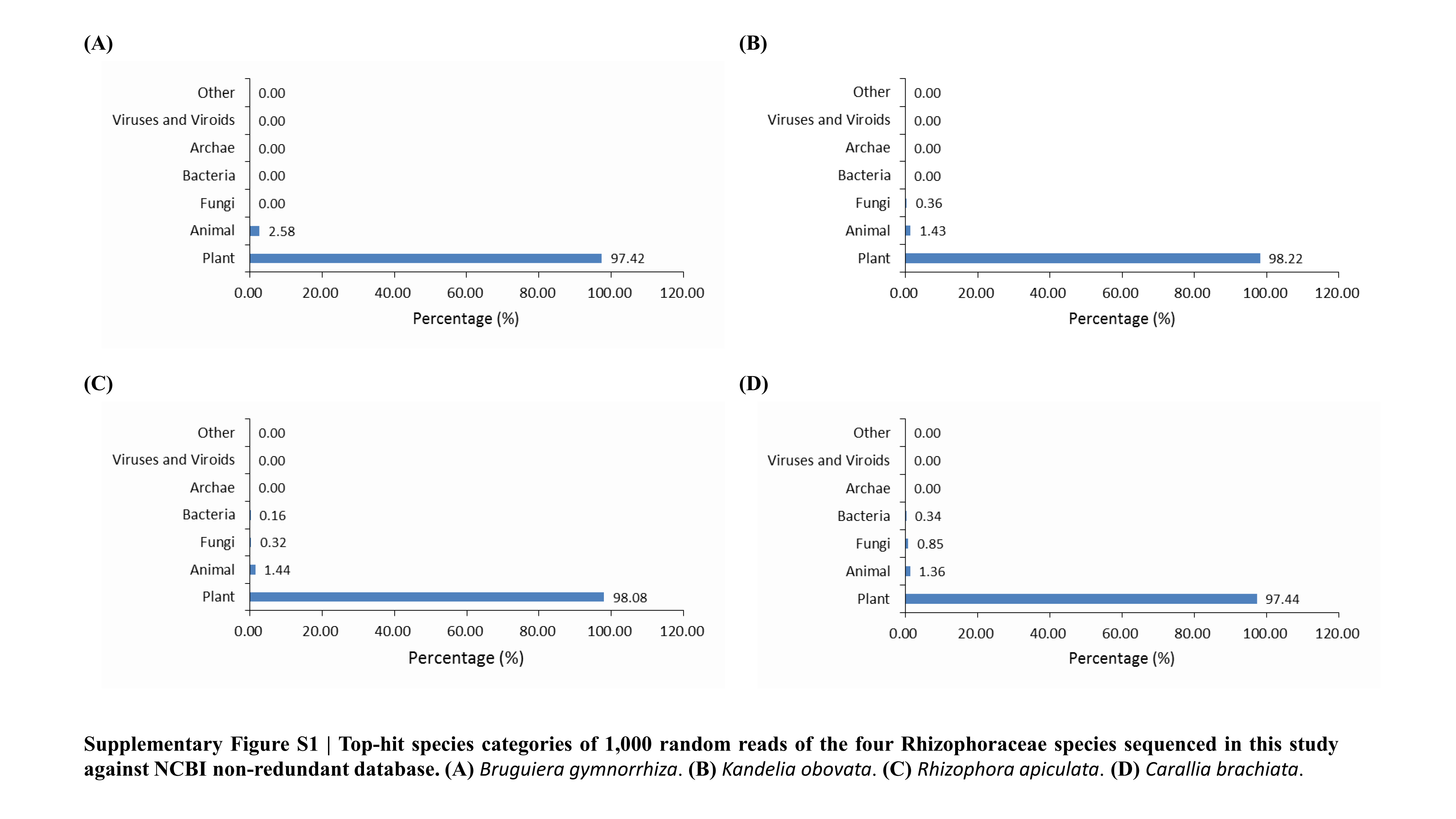

Supplement: Supplementary file 1 [file SupplementaryFigures1-9andTables1-6.ZIP › Figure_S1.tif]

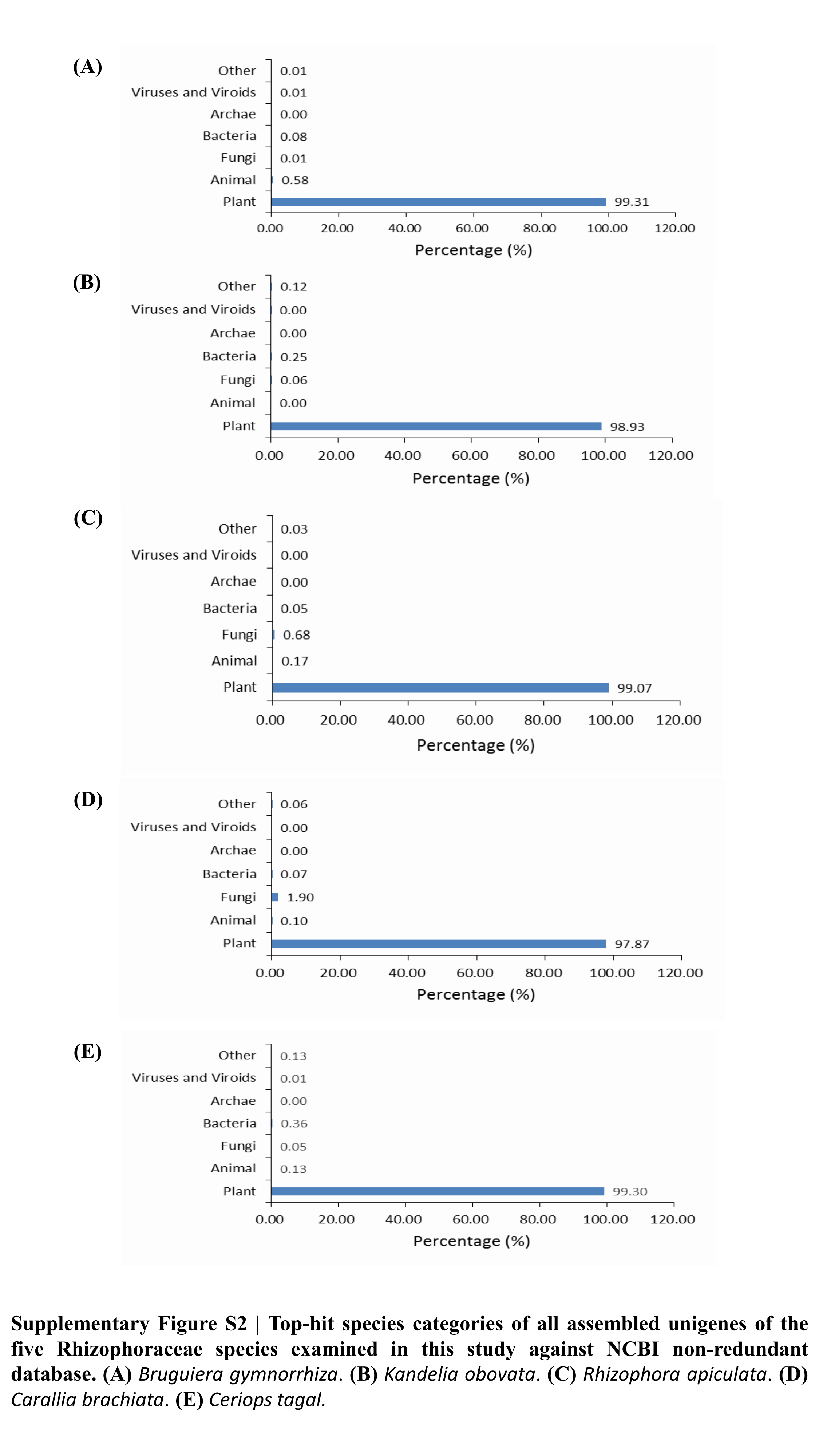

Supplement: Supplementary file 1 [file SupplementaryFigures1-9andTables1-6.ZIP › Figure_S2.tif]

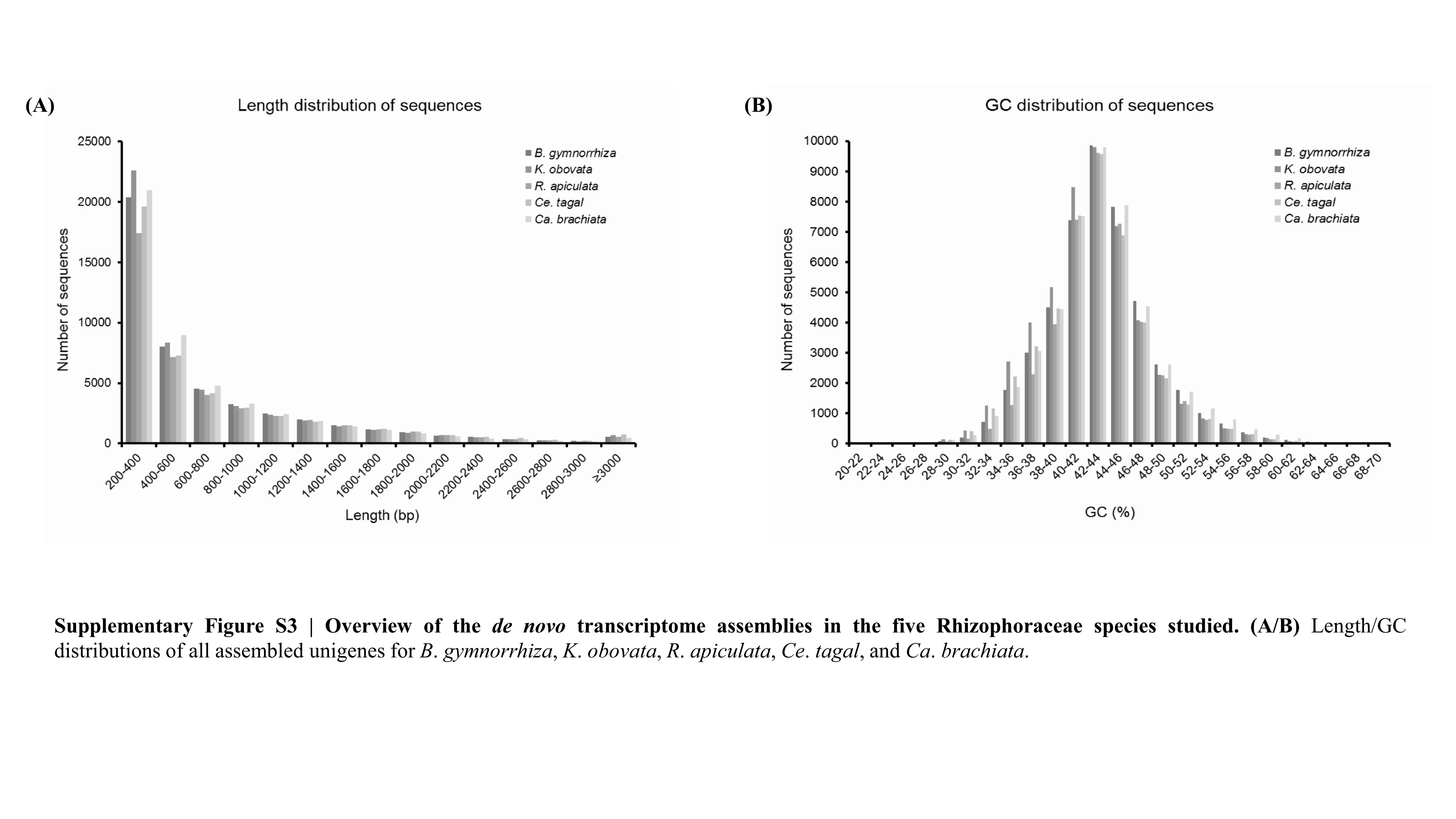

Supplement: Supplementary file 1 [file SupplementaryFigures1-9andTables1-6.ZIP › Figure_S3.tif]

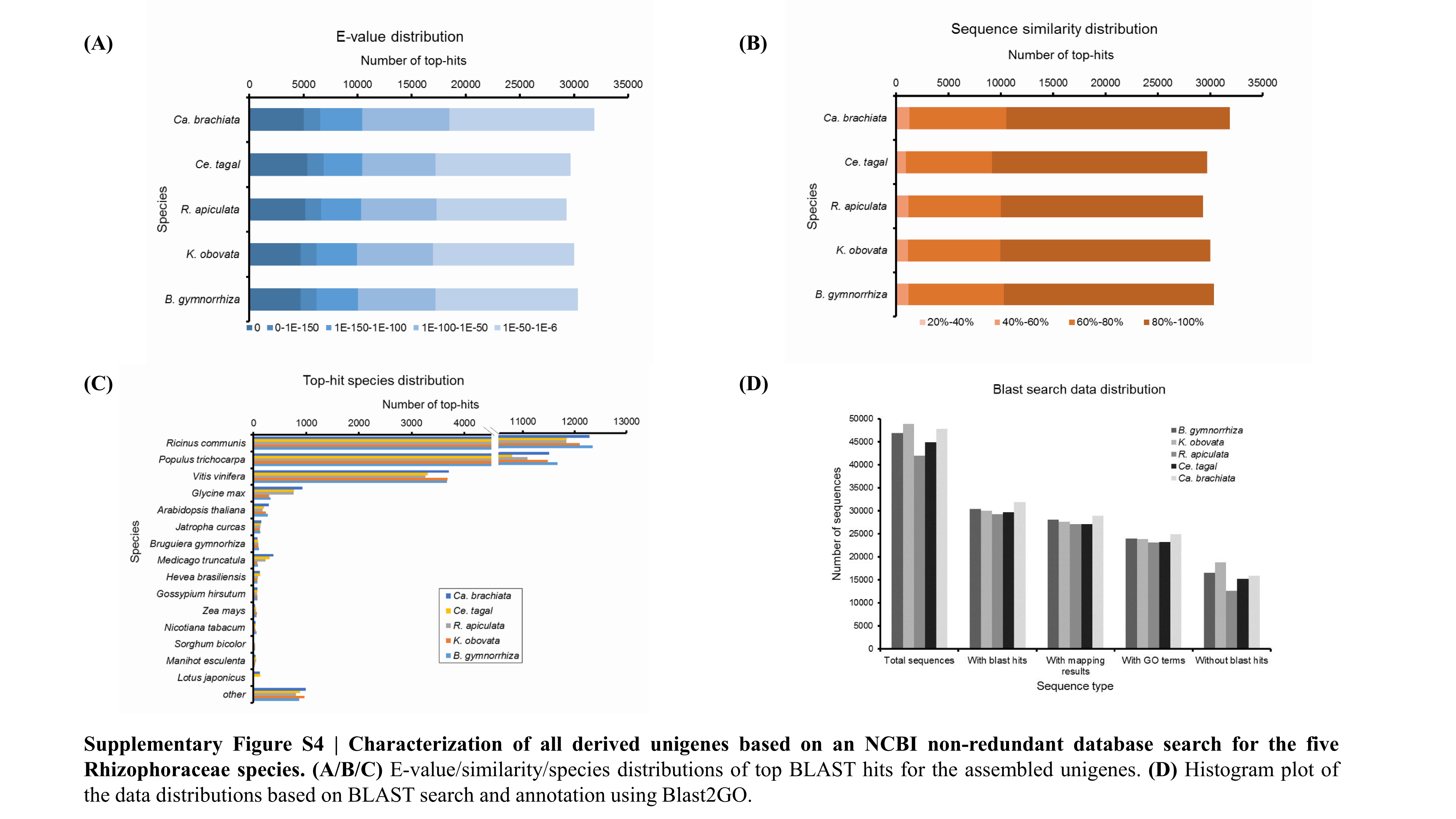

Supplement: Supplementary file 1 [file SupplementaryFigures1-9andTables1-6.ZIP › Figure_S4.tif]

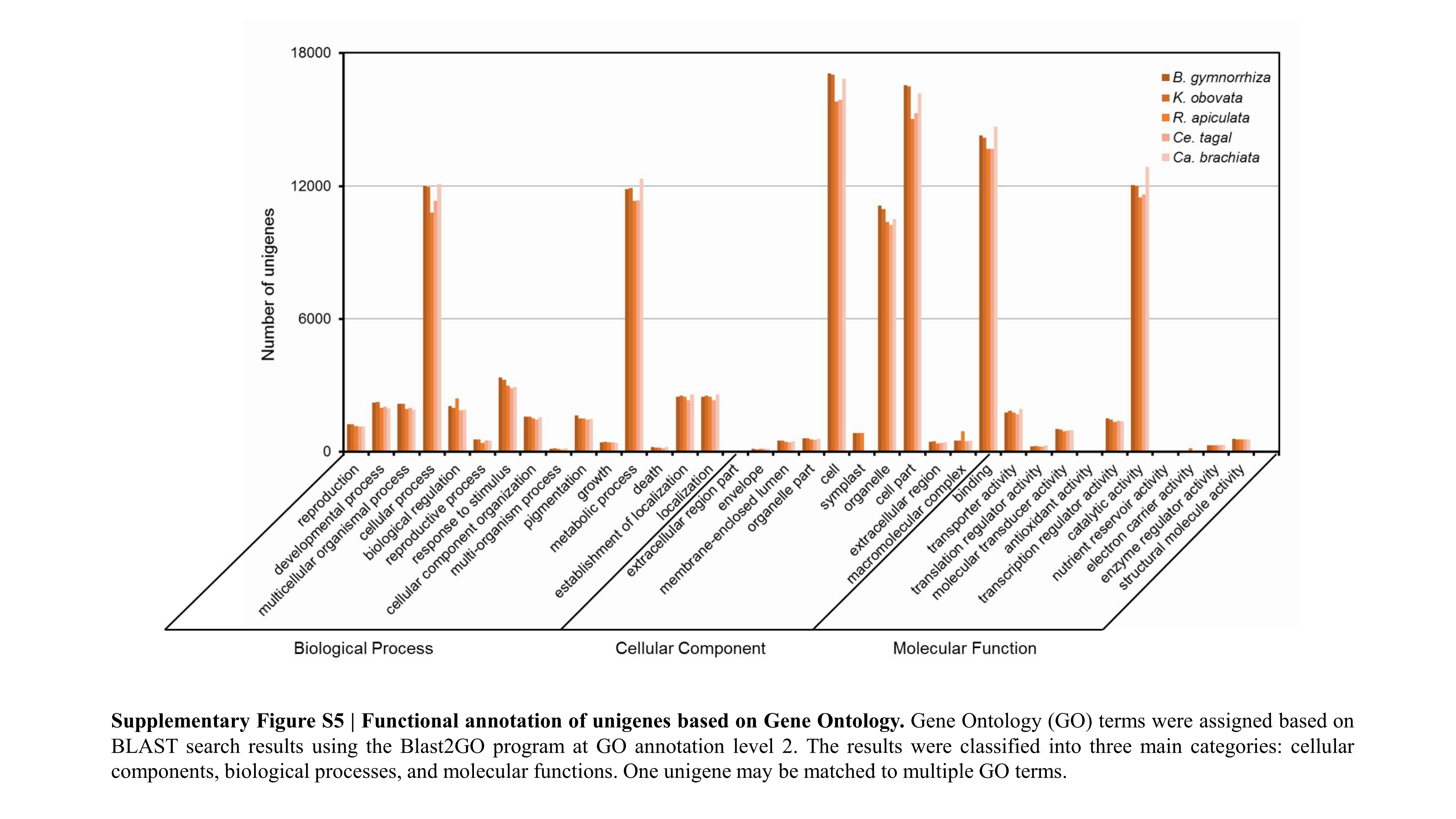

Supplement: Supplementary file 1 [file SupplementaryFigures1-9andTables1-6.ZIP › Figure_S5.tif]

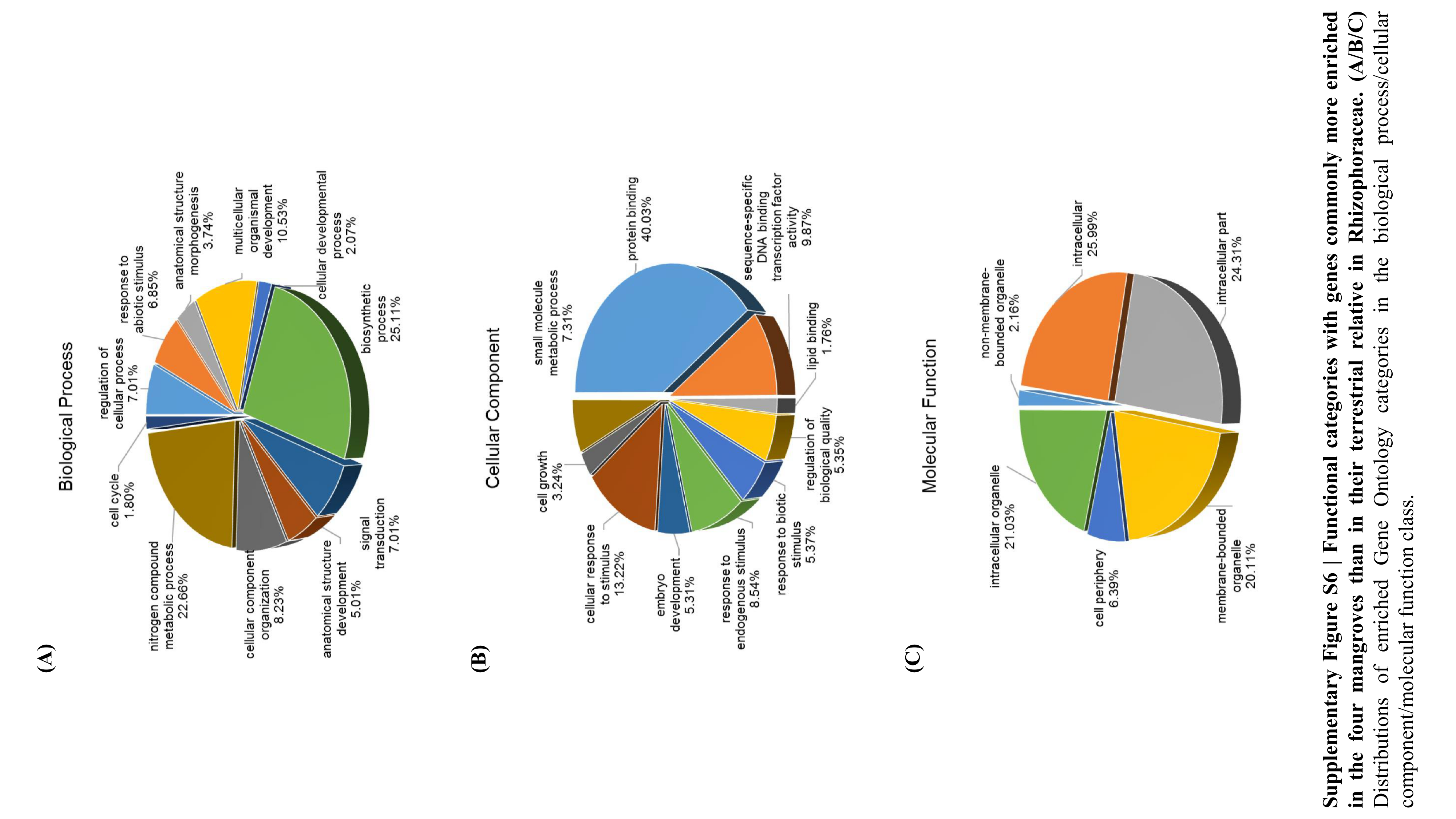

Supplement: Supplementary file 1 [file SupplementaryFigures1-9andTables1-6.ZIP › Figure_S6.tif]

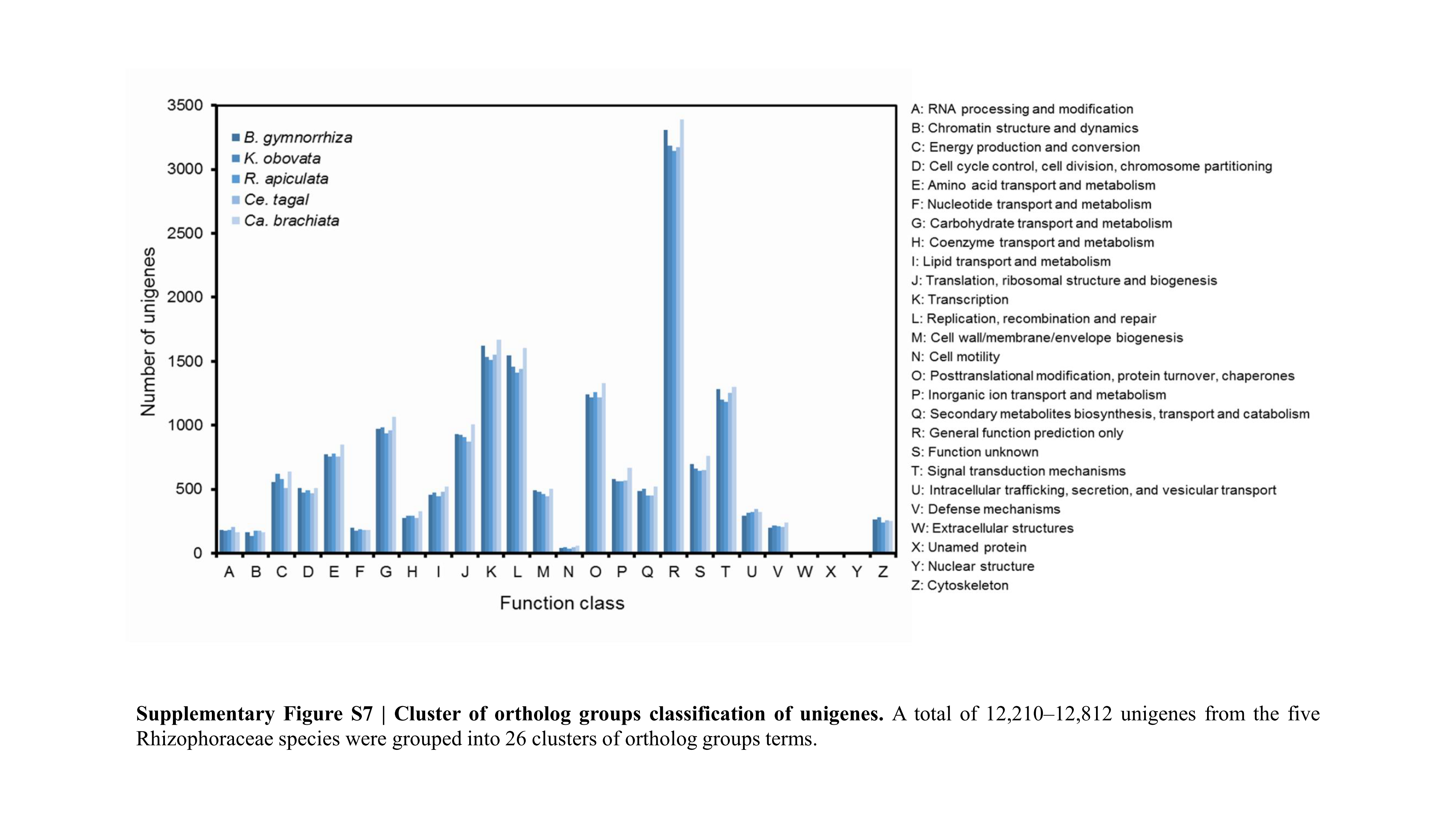

Supplement: Supplementary file 1 [file SupplementaryFigures1-9andTables1-6.ZIP › Figure_S7.tif]

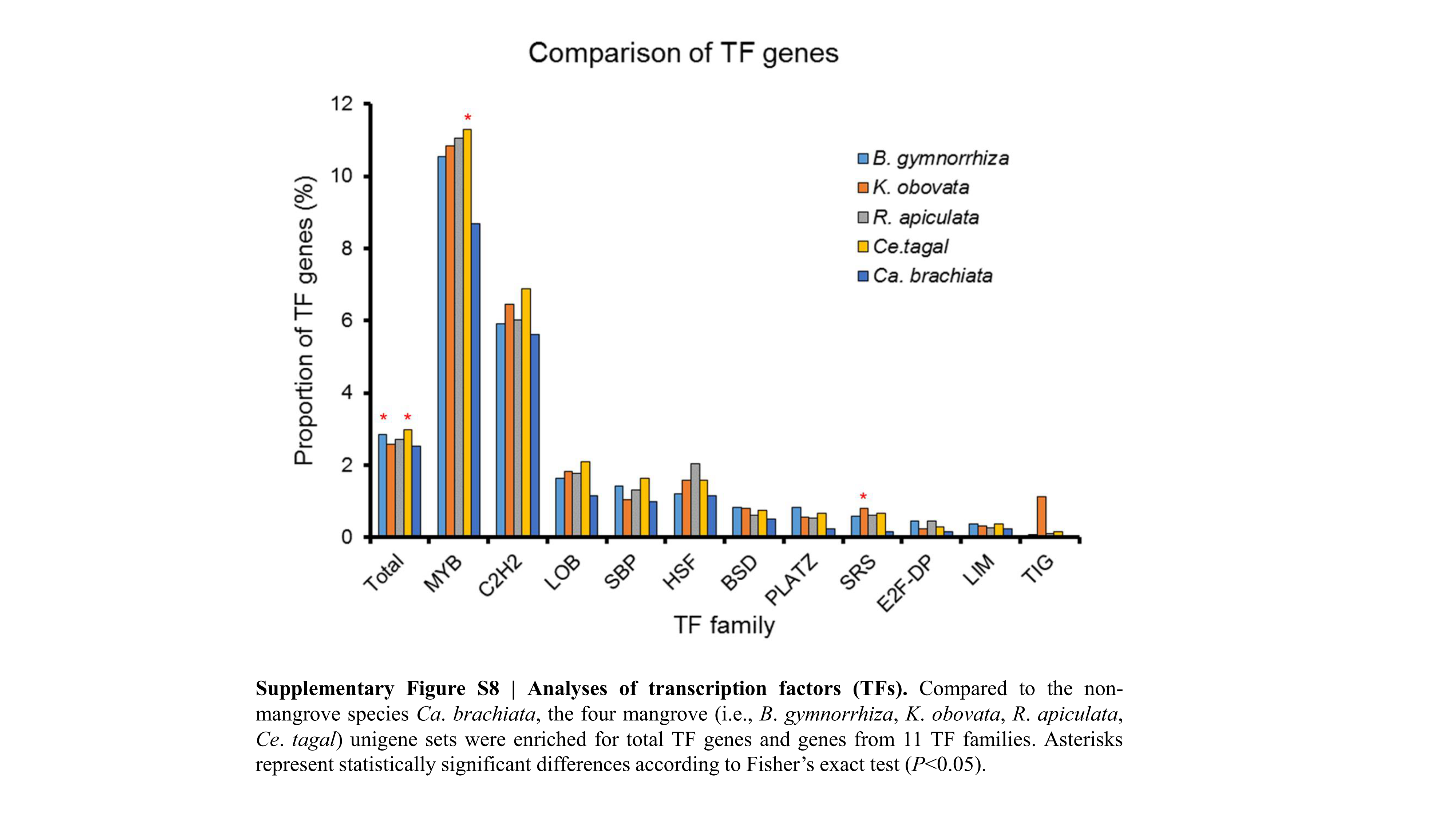

Supplement: Supplementary file 1 [file SupplementaryFigures1-9andTables1-6.ZIP › Figure_S8.tif]

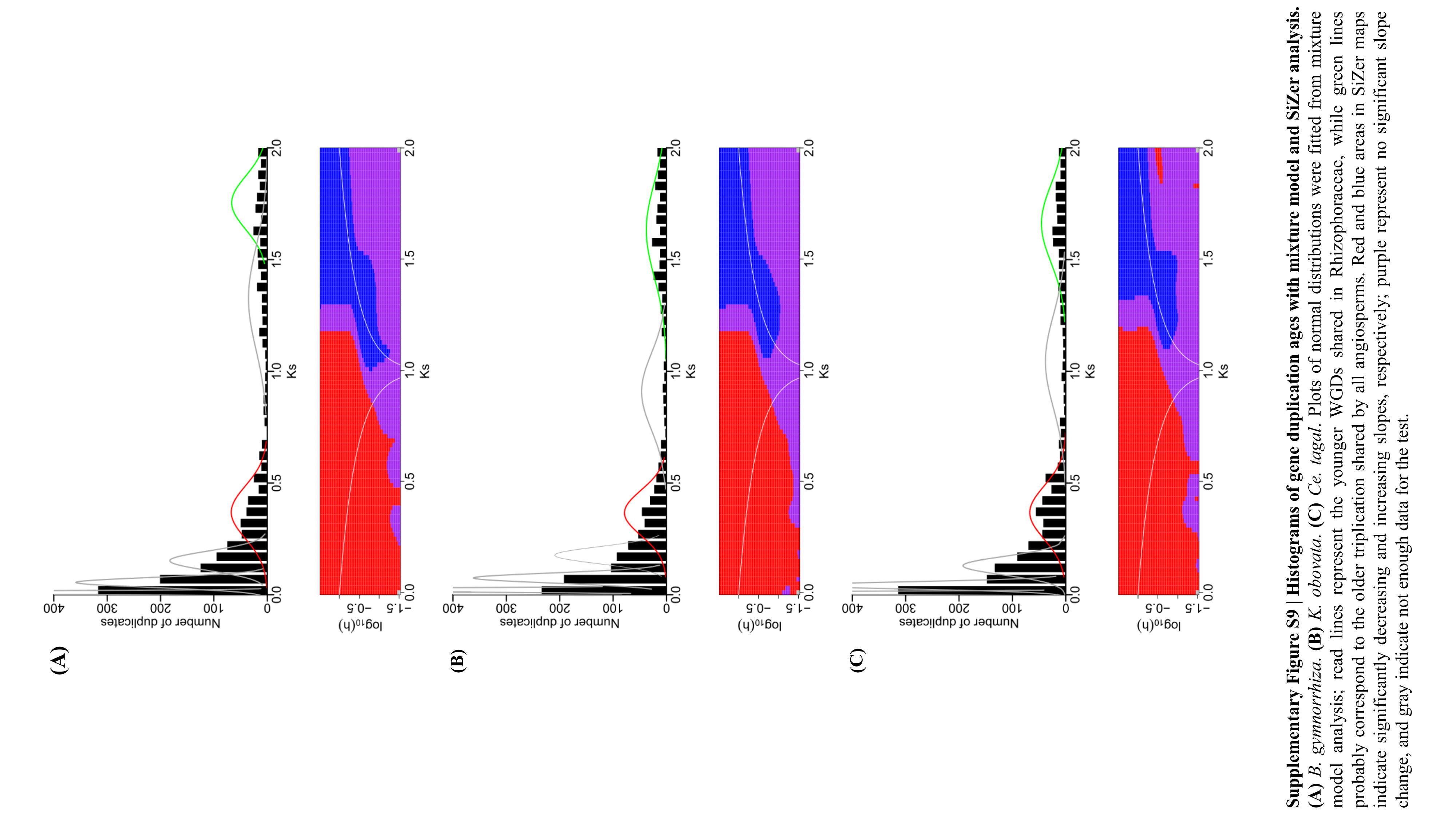

Supplement: Supplementary file 1 [file SupplementaryFigures1-9andTables1-6.ZIP › Figure_S9.tif]
