# Supplementary material for: Comparative Analysis of Transcriptomes in Rhizophoraceae Provides Insights into the Origin and Adaptive Evolution of Mangrove Plants in Intertidal Environments
Source: Front Plant Sci. 2017 May 16;8:795. doi: 10.3389/fpls.2017.00795 (PMC5432612; doi:10.3389/fpls.2017.00795)
Supplement: Supplementary file 2 [file SupplementaryTables7-14.ZIP › Supplementary_Table_S9.docx]

**Supplementary Table S9 | Summary statistics of cluster of ortholog groups (COG) classification and enrichment analysis.**

| COG class | Abbre-viation | Number of genes | | | | |  | *P*-value (Fisher’s exact test) | | | |
| --- | --- | --- | --- | --- | --- | --- | --- | --- | --- | --- | --- |
|  |  | bg | ko | ra | ct | cb |  | bg vs. cb | ko vs. cb | ra vs. cb | ct vs. cb |
| RNA processing and modification | A | 181 | 177 | 179 | 205 | 166 |  | 0.1764 | 0.1917 | 0.0928 | 0.0020 |
| Chromatin structure and dynamics | B | 167 | 137 | 173 | 177 | 163 |  | 0.4382 | 0.4500 | 0.1376 | 0.0800 |
| Energy production and conversion | C | 557 | 621 | 577 | 511 | 636 |  | 0.2242 | 0.3114 | 0.9062 | 0.0650 |
| Cell cycle control, cell division, chromosome partitioning | D | 512 | 473 | 492 | 467 | 512 |  | 0.3077 | 1.0000 | 0.3024 | 0.7691 |
| Amino acid transport and metabolism | E | 772 | 757 | 777 | 756 | 847 |  | 0.5380 | 0.5191 | 0.7194 | 0.9588 |
| Nucleotide transport and metabolism | F | 199 | 177 | 190 | 182 | 184 |  | 0.1805 | 0.7106 | 0.1923 | 0.3430 |
| Carbohydrate transport and metabolism | G | 970 | 981 | 938 | 960 | 1066 |  | 0.4603 | 0.9449 | 0.5923 | 0.8894 |
| Coenzyme transport and metabolism | H | 273 | 293 | 293 | 277 | 330 |  | 0.1175 | 0.6266 | 0.8710 | 0.4346 |
| Lipid transport and metabolism | I | 458 | 473 | 447 | 479 | 520 |  | 0.3123 | 0.8208 | 0.4704 | 0.6511 |
| Translation, ribosomal structure and biogenesis | J | 929 | 928 | 910 | 875 | 1005 |  | 0.7230 | 1.0000 | 0.9054 | 0.5330 |
| Transcription | K | 1624 | 1534 | 1509 | 1550 | 1667 |  | 0.2715 | 0.9398 | 0.8794 | 0.2575 |
| Replication, recombination and repair | L | 1547 | 1456 | 1412 | 1443 | 1605 |  | 0.4476 | 0.6168 | 0.4973 | 0.9077 |
| Cell wall/membrane/envelope biogenesis | M | 493 | 482 | 463 | 447 | 505 |  | 0.5397 | 0.6034 | 0.7930 | 0.8687 |
| Cell motility | N | 40 | 48 | 38 | 46 | 59 |  | 0.1304 | 0.5612 | 0.1261 | 0.4947 |
| Posttranslational modification, protein turnover, chaperones | O | 1242 | 1216 | 1258 | 1220 | 1329 |  | 0.9008 | 0.8184 | 0.1842 | 0.5172 |
| Inorganic ion transport and metabolism | P | 577 | 562 | 564 | 570 | 667 |  | 0.1385 | 0.1077 | 0.2665 | 0.4139 |
| Secondary metabolites biosynthesis, transport and catabolism | Q | 487 | 504 | 449 | 452 | 522 |  | 0.9233 | 0.4637 | 0.4713 | 0.6009 |
| General function prediction only | R | 3310 | 3182 | 3141 | 3171 | 3387 |  | 0.0618 | 0.4162 | 0.1735 | 0.0366 |
| Function unknown | S | 697 | 659 | 646 | 649 | 761 |  | 0.6276 | 0.2192 | 0.2604 | 0.3512 |
| Signal transduction mechanisms | T | 1280 | 1201 | 1184 | 1254 | 1302 |  | 0.2293 | 1.0000 | 0.8163 | 0.0523 |
| Intracellular trafficking, secretion, and vesicular transport | U | 293 | 319 | 324 | 347 | 324 |  | 0.6540 | 0.4242 | 0.1886 | 0.0188 |
| Defense mechanisms | V | 200 | 218 | 210 | 203 | 242 |  | 0.1793 | 0.8140 | 0.7040 | 0.5028 |
| Extracellular structures | W | 0 | 0 | 0 | 2 | 2 |  | 0.5005 | 0.5008 | 0.5013 | 1.0000 |
| Unamed protein | X | 0 | 0 | 0 | 0 | 0 |  | 1.0000 | 1.0000 | 1.0000 | 1.0000 |
| Nuclear structure | Y | 2 | 0 | 2 | 2 | 1 |  | 0.6136 | 1.0000 | 0.6065 | 0.6055 |
| Cytoskeleton | Z | 266 | 280 | 238 | 258 | 253 |  | 0.1986 | 0.0355 | 0.6483 | 0.1399 |

bg, ko, ra, ct and cb represent for *Bruguiera gymnorrhiza*, *Kandelia obovata*, *Rhizophora apiculata*, *Ceriops tagal* and *Carallia brachiata,* respectively.
