# Supplementary material for: Comparative Analysis of Transcriptomes in Rhizophoraceae Provides Insights into the Origin and Adaptive Evolution of Mangrove Plants in Intertidal Environments
Source: Front Plant Sci. 2017 May 16;8:795. doi: 10.3389/fpls.2017.00795 (PMC5432612; doi:10.3389/fpls.2017.00795)
Supplement: Supplementary file 2 [file SupplementaryTables7-14.ZIP › Supplementary_Table_S11.docx]

**Supplementary Table S11 | Comparison of transcription factors (TFs) among the five Rhizophoraceae species.**

| TF family | *B. gymnorrhiza* | *K. obovata* | *R. apiculata* | *Ce. tagal* | *Ca. brachiata* |
| --- | --- | --- | --- | --- | --- |
| MYB | 141 | 136 | 125 | 151* | 105 |
| AP2-EREBP | 112 | 90 | 90 | 80 | 97 |
| WRKY | 80 | 63 | 54 | 60 | 66 |
| C2H2 | 79 | 81 | 68 | 92 | 68 |
| NAC | 74 | 74 | 68 | 65 | 59 |
| bHLH | 72 | 52 | 59 | 76 | 63 |
| HB | 72 | 56 | 57 | 63 | 62 |
| C3H | 69 | 60 | 52 | 62 | 73 |
| bZIP | 59 | 47 | 45 | 53 | 50 |
| GRAS | 55 | 57 | 51 | 56 | 60 |
| mTERF | 41 | 7 | 26 | 41 | 31 |
| CCAAT | 38 | 31 | 35 | 33 | 37 |
| C2C2-GATA | 30 | 25 | 26 | 24 | 25 |
| ABI3VP1 | 30 | 25 | 26 | 22 | 21 |
| Trihelix | 29 | 2 | 28 | 34 | 26 |
| FAR1 | 25 | 27 | 24 | 27 | 23 |
| G2-like | 23 | 27 | 24 | 33 | 36 |
| LOB | 22 | 23 | 20 | 28 | 14 |
| C2C2-Dof | 21 | 25 | 19 | 27 | 32 |
| ARF | 19 | 16 | 20 | 28 | 21 |
| SBP | 19 | 13 | 15 | 22 | 12 |
| TCP | 18 | 21 | 18 | 28 | 19 |
| Tify | 18 | 30 | 12 | 18 | 18 |
| HSF | 16 | 20 | 23 | 21 | 14 |
| MADS | 14 | 16 | 9 | 14 | 12 |
| FHA | 13 | 13 | 16 | 16 | 12 |
| C2C2-YABBY | 12 | 5 | 6 | 10 | 11 |
| BSD | 11 | 10 | 7 | 10 | 6 |
| PLATZ | 11 | 7 | 6 | 9 | 3 |
| TUB | 9 | 19 | 7 | 21 | 20 |
| TAZ | 8 | 6 | 6 | 5 | 7 |
| OFP | 8 | 5 | 9 | 12 | 6 |
| SRS | 8 | 10* | 7 | 9 | 2 |
| BES1 | 7 | 5 | 5 | 6 | 6 |
| RWP-RK | 7 | 7 | 4 | 6 | 6 |
| Alfin-like | 6 | 7 | 4 | 10 | 8 |
| BBR/BPC | 6 | 6 | 4 | 7 | 8 |
| E2F-DP | 6 | 3 | 5 | 4 | 2 |
| CSD | 5 | 5 | 3 | 4 | 8 |
| zf-HD | 5 |  | 6 | 5 | 7 |
| CPP | 5 | 6 | 8 | 3 | 4 |
| LIM | 5 | 4 | 3 | 5 | 3 |
| Sigma70-like | 4 | 8 | 5 | 2 | 6 |
| ARR-B | 3 | 3 | 4 | 5 | 5 |
| C2C2-CO-like | 3 | 6 | 3 | 4 | 5 |
| DBP | 3 | 3 | 2 | 3 | 3 |
| ULT | 3 | 1 | 1 | 2 | 1 |
| GeBP | 2 | 3 | 4 | 5 | 5 |
| VOZ | 2 | 65 | 3 | 3 | 2 |
| PBF-2-like | 2 | 2 | 1 | 3 | 2 |
| HRT | 2 | 1 | 1 | 2 | 2 |
| GRF | 2 | 1 | 2 | 1 | 2 |
| CAMTA | 1 | 4 | 0 | 0 | 3 |
| S1Fa-like | 1 | 2 | 1 | 1 | 1 |
| TIG | 1 | 14 | 1 | 2 | 0 |
| EIL | 0 | 1 | 4 | 4 | 9 |
| Total | 1,337 | 1,256 | 1,132 | 1,337 | 1,209 |

**P*<0.05 by Fisher’s exact test when compared with the non-mangrove species *C. brachiata*.
