# Supplementary material for: Comparative Analysis of Transcriptomes in Rhizophoraceae Provides Insights into the Origin and Adaptive Evolution of Mangrove Plants in Intertidal Environments
Source: Front Plant Sci. 2017 May 16;8:795. doi: 10.3389/fpls.2017.00795 (PMC5432612; doi:10.3389/fpls.2017.00795)
Supplement: Supplementary file 2 [file SupplementaryTables7-14.ZIP › Supplementary_Table_S12.docx]

**Supplementary Table S12 | Functional categories of genes preferentially retained in duplicate compared with that returned to single copy following the whole-genome duplication (WGD) event for the five Rhizophoraceae species.**

| Term Name | GO ID | Term Type | Depth | #Paleologs | #Non-paleologs | *P*-value (Fisher's exact test) | Corrected *P*-value (FDR) |
| --- | --- | --- | --- | --- | --- | --- | --- |
| **A. *Bruguiera gymnorrhiza*** | | | | | | | |
| translation factor activity, nucleic acid binding | GO:0008135 | F | 5 | 12 | 10 | 0.0001 | 0.0093 |
| response to abiotic stimulus | GO:0009628 | P | 3 | 82 | 37 | 0.0001 | 0.0101 |
| translation | GO:0006412 | P | 5 | 44 | 22 | 0.0005 | 0.0302 |
| response to stimulus | GO:0050896 | P | 2 | 269 | 90 | 0.0026 | 0.0394 |
| macromolecular complex | GO:0032991 | C | 2 | 37 | 18 | 0.0026 | 0.0433 |
| response to stress | GO:0006950 | P | 3 | 138 | 52 | 0.0014 | 0.0466 |
| ribosome | GO:0005840 | C | 4 | 37 | 18 | 0.0026 | 0.0481 |
| transcription regulator activity | GO:0030528 | F | 2 | 50 | 23 | 0.0017 | 0.0491 |
| gene expression | GO:0010467 | P | 4 | 223 | 77 | 0.0021 | 0.0496 |
| ribonucleoprotein complex | GO:0030529 | C | 3 | 37 | 18 | 0.0026 | 0.0541 |
| response to biotic stimulus | GO:0009607 | P | 3 | 55 | 25 | 0.0014 | 0.0579 |
| cellular macromolecule biosynthetic process | GO:0034645 | P | 5 | 223 | 75 | 0.0052 | 0.0633 |
| macromolecule biosynthetic process | GO:0009059 | P | 4 | 223 | 75 | 0.0052 | 0.0681 |
| cellular biosynthetic process | GO:0044249 | P | 4 | 223 | 75 | 0.0052 | 0.0738 |
| pollination | GO:0009856 | P | 3 | 11 | 7 | 0.0098 | 0.1030 |
| multi-organism process | GO:0051704 | P | 2 | 11 | 7 | 0.0098 | 0.1099 |
| nucleus | GO:0005634 | C | 5 | 275 | 87 | 0.0170 | 0.1694 |
| intracellular organelle lumen | GO:0070013 | C | 4 | 43 | 17 | 0.0369 | 0.2710 |
| membrane-enclosed lumen | GO:0031974 | C | 2 | 43 | 17 | 0.0369 | 0.2833 |
| nuclear lumen | GO:0031981 | C | 5 | 43 | 17 | 0.0369 | 0.2968 |
| external encapsulating structure | GO:0030312 | C | 3 | 52 | 20 | 0.0335 | 0.2978 |
| tropism | GO:0009606 | P | 3 | 6 | 4 | 0.0440 | 0.3099 |
| organelle lumen | GO:0043233 | C | 3 | 43 | 17 | 0.0369 | 0.3116 |
| cell wall | GO:0005618 | C | 4 | 52 | 20 | 0.0335 | 0.3143 |
| carbohydrate binding | GO:0030246 | F | 3 | 14 | 7 | 0.0479 | 0.3235 |
| **B. *Kandelia obovata*** | | | | | | | |
| cytosol | GO:0005829 | C | 5 | 65 | 36 | 0.0000 | 0.0024 |
| embryo development | GO:0009790 | P | 3 | 29 | 16 | 0.0043 | 0.3305 |
| transcription regulator activity | GO:0030528 | F | 2 | 38 | 18 | 0.0189 | 0.9711 |
| **C. *Rhizophora apiculata*** | | | | | | | |
| response to endogenous stimulus | GO:0009719 | P | 3 | 42 | 32 | 0.0000 | 0.0000 |
| structural molecule activity | GO:0005198 | F | 2 | 37 | 27 | 0.0000 | 0.0001 |
| extracellular region | GO:0005576 | C | 2 | 17 | 13 | 0.0003 | 0.0205 |
| ribosome | GO:0005840 | C | 4 | 41 | 24 | 0.0007 | 0.0271 |
| ribonucleoprotein complex | GO:0030529 | C | 3 | 41 | 24 | 0.0007 | 0.0339 |
| cellular macromolecule biosynthetic process | GO:0034645 | P | 5 | 154 | 68 | 0.0022 | 0.0517 |
| macromolecule biosynthetic process | GO:0009059 | P | 4 | 154 | 68 | 0.0022 | 0.0591 |
| intracellular non-membrane-bounded organelle | GO:0043232 | C | 4 | 71 | 35 | 0.0033 | 0.0626 |
| cellular biosynthetic process | GO:0044249 | P | 4 | 154 | 68 | 0.0022 | 0.0689 |
| non-membrane-bounded organelle | GO:0043228 | C | 3 | 71 | 35 | 0.0033 | 0.0696 |
| macromolecular complex | GO:0032991 | C | 2 | 67 | 33 | 0.0044 | 0.0756 |
| gene expression | GO:0010467 | P | 4 | 158 | 68 | 0.0049 | 0.0762 |
| transcription, DNA-dependent | GO:0006351 | P | 5 | 113 | 50 | 0.0083 | 0.0822 |
| RNA biosynthetic process | GO:0032774 | P | 5 | 113 | 50 | 0.0083 | 0.0868 |
| RNA metabolic process | GO:0016070 | P | 5 | 113 | 50 | 0.0083 | 0.0919 |
| response to biotic stimulus | GO:0009607 | P | 3 | 24 | 14 | 0.0101 | 0.0952 |
| organelle organization | GO:0006996 | P | 5 | 9 | 7 | 0.0083 | 0.0973 |
| nucleus | GO:0005634 | C | 5 | 179 | 75 | 0.0071 | 0.1032 |
| cellular component organization or biogenesis at cellular level | GO:0071841 | P | 3 | 9 | 7 | 0.0083 | 0.1038 |
| mitochondrion organization | GO:0007005 | P | 6 | 0 | 4 | 0.0123 | 0.1099 |
| cellular component organization at cellular level | GO:0071842 | P | 4 | 9 | 7 | 0.0083 | 0.1112 |
| nucleic acid metabolic process | GO:0090304 | P | 5 | 130 | 55 | 0.0169 | 0.1442 |
| nucleic acid binding transcription factor activity | GO:0001071 | F | 2 | 55 | 26 | 0.0210 | 0.1643 |
| sequence-specific DNA binding transcription factor activity | GO:0003700 | F | 3 | 55 | 26 | 0.0210 | 0.1715 |
| signal transduction | GO:0007165 | P | 3 | 82 | 36 | 0.0274 | 0.1909 |
| cellular response to stimulus | GO:0051716 | P | 3 | 82 | 36 | 0.0274 | 0.1982 |
| regulation of cellular process | GO:0050794 | P | 3 | 82 | 36 | 0.0274 | 0.2062 |
| DNA binding | GO:0003677 | F | 4 | 96 | 41 | 0.0316 | 0.2124 |
| signaling | GO:0023052 | P | 2 | 83 | 36 | 0.0335 | 0.2170 |
| chromosome | GO:0005694 | C | 5 | 2 | 3 | 0.0372 | 0.2331 |
| **D. *Ceriops tagal*** |  |  |  |  |  |  |  |
| nucleic acid binding transcription factor activity | GO:0001071 | F | 2 | 50 | 44 | 0.0000 | 0.0000 |
| sequence-specific DNA binding transcription factor activity | GO:0003700 | F | 3 | 50 | 44 | 0.0000 | 0.0000 |
| DNA binding | GO:0003677 | F | 4 | 99 | 67 | 0.0000 | 0.0000 |
| hydrolase activity, acting on ester bonds | GO:0016788 | F | 4 | 10 | 12 | 0.0000 | 0.0006 |
| nuclease activity | GO:0004518 | F | 5 | 10 | 12 | 0.0000 | 0.0007 |
| enzyme regulator activity | GO:0030234 | F | 2 | 10 | 11 | 0.0000 | 0.0012 |
| transcription, DNA-dependent | GO:0006351 | P | 5 | 133 | 74 | 0.0001 | 0.0027 |
| RNA biosynthetic process | GO:0032774 | P | 5 | 133 | 74 | 0.0001 | 0.0031 |
| RNA metabolic process | GO:0016070 | P | 5 | 133 | 74 | 0.0001 | 0.0035 |
| gene expression | GO:0010467 | P | 4 | 175 | 92 | 0.0004 | 0.0062 |
| nucleic acid binding | GO:0003676 | F | 3 | 185 | 95 | 0.0009 | 0.0112 |
| nucleic acid metabolic process | GO:0090304 | P | 5 | 156 | 82 | 0.0008 | 0.0112 |
| regulation of biological quality | GO:0065008 | P | 3 | 28 | 20 | 0.0008 | 0.0121 |
| cellular macromolecule biosynthetic process | GO:0034645 | P | 5 | 169 | 86 | 0.0022 | 0.0235 |
| macromolecule biosynthetic process | GO:0009059 | P | 4 | 169 | 86 | 0.0022 | 0.0251 |
| cellular biosynthetic process | GO:0044249 | P | 4 | 169 | 86 | 0.0022 | 0.0269 |
| response to stimulus | GO:0050896 | P | 2 | 227 | 110 | 0.0048 | 0.0469 |
| cell communication | GO:0007154 | P | 3 | 8 | 7 | 0.0089 | 0.0832 |
| homeostatic process | GO:0042592 | P | 4 | 10 | 8 | 0.0129 | 0.1083 |
| cellular homeostasis | GO:0019725 | P | 3 | 10 | 8 | 0.0129 | 0.1140 |
| growth | GO:0040007 | P | 2 | 26 | 16 | 0.0229 | 0.1605 |
| signal transducer activity | GO:0004871 | F | 3 | 53 | 29 | 0.0225 | 0.1644 |
| molecular transducer activity | GO:0060089 | F | 2 | 53 | 29 | 0.0225 | 0.1718 |
| transporter activity | GO:0005215 | F | 2 | 90 | 46 | 0.0221 | 0.1769 |
| receptor activity | GO:0004872 | F | 4 | 33 | 19 | 0.0329 | 0.2212 |
| **E. *Carallia brachiata*** |  |  |  |  |  |  |  |
| cell differentiation | GO:0030154 | P | 4 | 18 | 15 | 0.0000 | 0.0003 |
| cellular developmental process | GO:0048869 | P | 3 | 18 | 15 | 0.0000 | 0.0007 |
| hydrolase activity, acting on ester bonds | GO:0016788 | F | 4 | 8 | 7 | 0.0013 | 0.0261 |
| endoplasmic reticulum | GO:0005783 | C | 5 | 26 | 16 | 0.0008 | 0.0264 |
| anatomical structure development | GO:0048856 | P | 3 | 53 | 27 | 0.0010 | 0.0274 |
| death | GO:0016265 | P | 2 | 6 | 6 | 0.0007 | 0.0296 |
| nuclease activity | GO:0004518 | F | 5 | 8 | 7 | 0.0013 | 0.0298 |
| cell death | GO:0008219 | P | 3 | 6 | 6 | 0.0007 | 0.0394 |
| lipid binding | GO:0008289 | F | 3 | 5 | 5 | 0.0024 | 0.0439 |
| anatomical structure morphogenesis | GO:0009653 | P | 3 | 31 | 17 | 0.0033 | 0.0532 |
| cell cycle | GO:0007049 | P | 3 | 12 | 8 | 0.0095 | 0.1398 |
| developmental process | GO:0032502 | P | 2 | 126 | 47 | 0.0441 | 0.5948 |

F, P, and C refer to ‘molecular function’, ‘biological process’, and ‘cellular component’, respectively.
