# Supplementary material for: Comparative Analysis of Transcriptomes in Rhizophoraceae Provides Insights into the Origin and Adaptive Evolution of Mangrove Plants in Intertidal Environments
Source: Front Plant Sci. 2017 May 16;8:795. doi: 10.3389/fpls.2017.00795 (PMC5432612; doi:10.3389/fpls.2017.00795)
Supplement: Supplementary file 2 [file SupplementaryTables7-14.ZIP › Supplementary_Table_S13.docx]

**Supplementary Table S13 | Functional categories of genes commonly overrepresented in paleologs of the four mangroves compared with that in their terrestrial relative in Rhizophoraceae.**

| Term Name | GO ID | Term Type | Depth | #Mangrove paleologs | #Non-mangrove paleologs | *P*-value (Fisher's exact test) | Corrected *P*-value (FDR) |
| --- | --- | --- | --- | --- | --- | --- | --- |
| **A. *Bruguiera gymnorrhiza* vs. *Carallia brachiata*** | | | | | | | |
| binding | GO:0005488 | F | 2 | 222 | 237 | 0.0000 | 0.0000 |
| organelle | GO:0043226 | C | 2 | 170 | 179 | 0.0000 | 0.0000 |
| intracellular organelle | GO:0043229 | C | 3 | 170 | 179 | 0.0000 | 0.0000 |
| intracellular membrane-bounded organelle | GO:0043231 | C | 4 | 159 | 170 | 0.0000 | 0.0000 |
| membrane-bounded organelle | GO:0043227 | C | 3 | 159 | 170 | 0.0000 | 0.0000 |
| cellular process | GO:0009987 | P | 2 | 208 | 207 | 0.0000 | 0.0000 |
| cell part | GO:0044464 | C | 2 | 269 | 262 | 0.0000 | 0.0000 |
| cell | GO:0005623 | C | 2 | 277 | 269 | 0.0000 | 0.0000 |
| cellular metabolic process | GO:0044237 | P | 3 | 126 | 133 | 0.0000 | 0.0000 |
| intracellular part | GO:0044424 | C | 3 | 201 | 198 | 0.0000 | 0.0000 |
| cellular_component | GO:0005575 | C | 1 | 279 | 269 | 0.0000 | 0.0000 |
| response to stimulus | GO:0050896 | P | 2 | 68 | 90 | 0.0000 | 0.0000 |
| nucleus | GO:0005634 | C | 5 | 69 | 87 | 0.0000 | 0.0000 |
| protein binding | GO:0005515 | F | 3 | 66 | 85 | 0.0000 | 0.0000 |
| intracellular | GO:0005622 | C | 3 | 224 | 216 | 0.0000 | 0.0001 |
| biological_process | GO:0008150 | P | 1 | 280 | 267 | 0.0000 | 0.0001 |
| cell periphery | GO:0071944 | C | 3 | 56 | 70 | 0.0001 | 0.0005 |
| gene expression | GO:0010467 | P | 4 | 73 | 77 | 0.0001 | 0.0008 |
| cellular macromolecule biosynthetic process | GO:0034645 | P | 5 | 72 | 75 | 0.0001 | 0.0010 |
| macromolecule biosynthetic process | GO:0009059 | P | 4 | 72 | 75 | 0.0001 | 0.0010 |
| nucleobase, nucleoside, nucleotide and nucleic acid metabolic process | GO:0006139 | P | 4 | 69 | 73 | 0.0001 | 0.0010 |
| cellular biosynthetic process | GO:0044249 | P | 4 | 72 | 75 | 0.0001 | 0.0011 |
| plasma membrane | GO:0005886 | C | 4 | 50 | 60 | 0.0004 | 0.0027 |
| multicellular organismal process | GO:0032501 | P | 2 | 44 | 57 | 0.0004 | 0.0028 |
| cellular macromolecule metabolic process | GO:0044260 | P | 4 | 107 | 105 | 0.0004 | 0.0028 |
| biosynthetic process | GO:0009058 | P | 3 | 106 | 104 | 0.0004 | 0.0029 |
| developmental process | GO:0032502 | P | 2 | 47 | 57 | 0.0006 | 0.0035 |
| response to stress | GO:0006950 | P | 3 | 38 | 52 | 0.0008 | 0.0046 |
| multicellular organismal development | GO:0007275 | P | 3 | 44 | 54 | 0.0009 | 0.0050 |
| cellular protein metabolic process | GO:0044267 | P | 5 | 48 | 52 | 0.0020 | 0.0114 |
| transcription, DNA-dependent | GO:0006351 | P | 5 | 53 | 53 | 0.0027 | 0.0138 |
| RNA biosynthetic process | GO:0032774 | P | 5 | 53 | 53 | 0.0027 | 0.0142 |
| RNA metabolic process | GO:0016070 | P | 5 | 53 | 53 | 0.0027 | 0.0147 |
| molecular_function | GO:0003674 | F | 1 | 343 | 318 | 0.0046 | 0.0229 |
| response to abiotic stimulus | GO:0009628 | P | 3 | 21 | 37 | 0.0057 | 0.0275 |
| biological regulation | GO:0065007 | P | 2 | 34 | 39 | 0.0095 | 0.0444 |
| cellular component organization or biogenesis | GO:0071840 | P | 2 | 35 | 39 | 0.0102 | 0.0453 |
| cellular component organization | GO:0016043 | P | 3 | 35 | 39 | 0.0102 | 0.0465 |
| nucleotide binding | GO:0000166 | F | 3 | 59 | 58 | 0.0112 | 0.0472 |
| membrane | GO:0016020 | C | 3 | 103 | 99 | 0.0112 | 0.0484 |
| DNA binding | GO:0003677 | F | 4 | 37 | 38 | 0.0143 | 0.0588 |
| reproduction | GO:0000003 | P | 2 | 23 | 32 | 0.0177 | 0.0712 |
| mitochondrion | GO:0005739 | C | 5 | 27 | 31 | 0.0269 | 0.1057 |
| nucleic acid binding transcription factor activity | GO:0001071 | F | 2 | 29 | 31 | 0.0302 | 0.1134 |
| sequence-specific DNA binding transcription factor activity | GO:0003700 | F | 3 | 29 | 31 | 0.0302 | 0.1159 |
| response to biotic stimulus | GO:0009607 | P | 3 | 12 | 25 | 0.0364 | 0.1282 |
| intracellular non-membrane-bounded organelle | GO:0043232 | C | 4 | 26 | 29 | 0.0361 | 0.1297 |
| non-membrane-bounded organelle | GO:0043228 | C | 3 | 26 | 29 | 0.0361 | 0.1325 |
| post-embryonic development | GO:0009791 | P | 3 | 21 | 27 | 0.0393 | 0.1355 |
| transporter activity | GO:0005215 | F | 2 | 22 | 27 | 0.0413 | 0.1396 |
| response to endogenous stimulus | GO:0009719 | P | 3 | 15 | 24 | 0.0497 | 0.1647 |
| **B. *Kandelia obovata* vs. *Carallia brachiata*** | | | | | | | |
| cellular metabolic process | GO:0044237 | P | 3 | 126 | 125 | 0.0000 | 0.0000 |
| binding | GO:0005488 | F | 2 | 222 | 209 | 0.0000 | 0.0000 |
| protein binding | GO:0005515 | F | 3 | 66 | 75 | 0.0000 | 0.0000 |
| cell periphery | GO:0071944 | C | 3 | 56 | 63 | 0.0000 | 0.0000 |
| cytoplasm | GO:0005737 | C | 4 | 154 | 145 | 0.0000 | 0.0000 |
| primary metabolic process | GO:0044238 | P | 3 | 173 | 161 | 0.0000 | 0.0000 |
| nucleotide binding | GO:0000166 | F | 3 | 59 | 59 | 0.0000 | 0.0001 |
| intracellular part | GO:0044424 | C | 3 | 201 | 184 | 0.0000 | 0.0001 |
| cellular process | GO:0009987 | P | 2 | 208 | 189 | 0.0000 | 0.0003 |
| cellular protein metabolic process | GO:0044267 | P | 5 | 48 | 51 | 0.0000 | 0.0003 |
| cytoplasmic part | GO:0044444 | C | 4 | 120 | 113 | 0.0000 | 0.0003 |
| plasma membrane | GO:0005886 | C | 4 | 50 | 50 | 0.0000 | 0.0004 |
| kinase activity | GO:0016301 | F | 5 | 36 | 44 | 0.0001 | 0.0007 |
| transferase activity, transferring phosphorus-containing groups | GO:0016772 | F | 4 | 36 | 44 | 0.0001 | 0.0008 |
| multicellular organismal process | GO:0032501 | P | 2 | 44 | 44 | 0.0001 | 0.0012 |
| multicellular organismal development | GO:0007275 | P | 3 | 44 | 44 | 0.0001 | 0.0013 |
| cytosol | GO:0005829 | C | 5 | 19 | 36 | 0.0002 | 0.0020 |
| developmental process | GO:0032502 | P | 2 | 47 | 46 | 0.0008 | 0.0062 |
| biological regulation | GO:0065007 | P | 2 | 34 | 35 | 0.0008 | 0.0064 |
| cellular macromolecule metabolic process | GO:0044260 | P | 4 | 107 | 99 | 0.0008 | 0.0067 |
| regulation of biological process | GO:0050789 | P | 2 | 28 | 31 | 0.0016 | 0.0120 |
| post-embryonic development | GO:0009791 | P | 3 | 21 | 28 | 0.0025 | 0.0176 |
| signal transduction | GO:0007165 | P | 3 | 25 | 28 | 0.0031 | 0.0183 |
| cellular response to stimulus | GO:0051716 | P | 3 | 25 | 28 | 0.0031 | 0.0191 |
| signaling | GO:0023052 | P | 2 | 25 | 28 | 0.0031 | 0.0199 |
| regulation of cellular process | GO:0050794 | P | 3 | 25 | 28 | 0.0031 | 0.0207 |
| intracellular | GO:0005622 | C | 3 | 224 | 196 | 0.0048 | 0.0274 |
| biological_process | GO:0008150 | P | 1 | 280 | 242 | 0.0062 | 0.0339 |
| protein metabolic process | GO:0019538 | P | 4 | 70 | 65 | 0.0065 | 0.0345 |
| macromolecule metabolic process | GO:0043170 | P | 3 | 126 | 113 | 0.0073 | 0.0375 |
| reproduction | GO:0000003 | P | 2 | 23 | 24 | 0.0079 | 0.0392 |
| external encapsulating structure | GO:0030312 | C | 3 | 8 | 21 | 0.0092 | 0.0444 |
| cell wall | GO:0005618 | C | 4 | 8 | 20 | 0.0120 | 0.0560 |
| cellular macromolecule biosynthetic process | GO:0034645 | P | 5 | 72 | 66 | 0.0141 | 0.0604 |
| macromolecule biosynthetic process | GO:0009059 | P | 4 | 72 | 66 | 0.0141 | 0.0621 |
| cellular biosynthetic process | GO:0044249 | P | 4 | 72 | 66 | 0.0141 | 0.0640 |
| response to endogenous stimulus | GO:0009719 | P | 3 | 15 | 20 | 0.0155 | 0.0646 |
| nucleus | GO:0005634 | C | 5 | 69 | 63 | 0.0210 | 0.0828 |
| endoplasmic reticulum | GO:0005783 | C | 5 | 16 | 19 | 0.0206 | 0.0834 |
| translation | GO:0006412 | P | 5 | 19 | 19 | 0.0229 | 0.0880 |
| transcription regulator activity | GO:0030528 | F | 2 | 13 | 18 | 0.0237 | 0.0891 |
| macromolecular complex | GO:0032991 | C | 2 | 14 | 17 | 0.0312 | 0.1022 |
| ribosome | GO:0005840 | C | 4 | 14 | 17 | 0.0312 | 0.1044 |
| embryo development | GO:0009790 | P | 3 | 8 | 16 | 0.0331 | 0.1063 |
| ribonucleoprotein complex | GO:0030529 | C | 3 | 14 | 17 | 0.0312 | 0.1067 |
| organelle | GO:0043226 | C | 2 | 170 | 148 | 0.0302 | 0.1080 |
| gene expression | GO:0010467 | P | 4 | 73 | 66 | 0.0309 | 0.1082 |
| intracellular organelle | GO:0043229 | C | 3 | 170 | 148 | 0.0302 | 0.1106 |
| vacuole | GO:0005773 | C | 5 | 4 | 15 | 0.0379 | 0.1191 |
| protein modification process | GO:0006464 | P | 5 | 34 | 32 | 0.0404 | 0.1221 |
| macromolecule modification | GO:0043412 | P | 4 | 34 | 32 | 0.0404 | 0.1245 |
| intracellular membrane-bounded organelle | GO:0043231 | C | 4 | 159 | 138 | 0.0471 | 0.1369 |
| membrane-bounded organelle | GO:0043227 | C | 3 | 159 | 138 | 0.0471 | 0.1395 |
| **C. *Rhizophora apiculata* vs. *Carallia brachiata*** | | | | | | | |
| organelle | GO:0043226 | C | 2 | 170 | 169 | 0.0000 | 0.0000 |
| cytoplasmic part | GO:0044444 | C | 4 | 120 | 120 | 0.0000 | 0.0000 |
| intracellular organelle | GO:0043229 | C | 3 | 170 | 165 | 0.0000 | 0.0000 |
| response to stimulus | GO:0050896 | P | 2 | 68 | 74 | 0.0000 | 0.0001 |
| nucleus | GO:0005634 | C | 5 | 69 | 75 | 0.0000 | 0.0001 |
| cell periphery | GO:0071944 | C | 3 | 56 | 64 | 0.0000 | 0.0003 |
| biological regulation | GO:0065007 | P | 2 | 34 | 53 | 0.0000 | 0.0007 |
| plasma membrane | GO:0005886 | C | 4 | 50 | 56 | 0.0001 | 0.0012 |
| regulation of biological process | GO:0050789 | P | 2 | 28 | 46 | 0.0001 | 0.0024 |
| establishment of localization | GO:0051234 | P | 2 | 36 | 46 | 0.0002 | 0.0036 |
| localization | GO:0051179 | P | 2 | 36 | 46 | 0.0002 | 0.0039 |
| transport | GO:0006810 | P | 3 | 36 | 46 | 0.0002 | 0.0043 |
| DNA binding | GO:0003677 | F | 4 | 37 | 41 | 0.0009 | 0.0136 |
| protein binding | GO:0005515 | F | 3 | 66 | 64 | 0.0016 | 0.0154 |
| response to stress | GO:0006950 | P | 3 | 38 | 39 | 0.0017 | 0.0156 |
| signal transduction | GO:0007165 | P | 3 | 25 | 36 | 0.0014 | 0.0157 |
| macromolecular complex | GO:0032991 | C | 2 | 14 | 33 | 0.0015 | 0.0160 |
| cellular response to stimulus | GO:0051716 | P | 3 | 25 | 36 | 0.0014 | 0.0166 |
| intracellular non-membrane-bounded organelle | GO:0043232 | C | 4 | 26 | 35 | 0.0019 | 0.0166 |
| response to endogenous stimulus | GO:0009719 | P | 3 | 15 | 32 | 0.0021 | 0.0173 |
| non-membrane-bounded organelle | GO:0043228 | C | 3 | 26 | 35 | 0.0019 | 0.0174 |
| signaling | GO:0023052 | P | 2 | 25 | 36 | 0.0014 | 0.0177 |
| regulation of cellular process | GO:0050794 | P | 3 | 25 | 36 | 0.0014 | 0.0190 |
| transporter activity | GO:0005215 | F | 2 | 22 | 29 | 0.0065 | 0.0511 |
| structural molecule activity | GO:0005198 | F | 2 | 18 | 27 | 0.0084 | 0.0634 |
| cellular macromolecule biosynthetic process | GO:0034645 | P | 5 | 72 | 68 | 0.0113 | 0.0663 |
| intracellular membrane-bounded organelle | GO:0043231 | C | 4 | 159 | 145 | 0.0097 | 0.0674 |
| macromolecule biosynthetic process | GO:0009059 | P | 4 | 72 | 68 | 0.0113 | 0.0684 |
| response to abiotic stimulus | GO:0009628 | P | 3 | 21 | 26 | 0.0123 | 0.0698 |
| membrane-bounded organelle | GO:0043227 | C | 3 | 159 | 145 | 0.0097 | 0.0700 |
| cellular biosynthetic process | GO:0044249 | P | 4 | 72 | 68 | 0.0113 | 0.0707 |
| catabolic process | GO:0009056 | P | 3 | 19 | 26 | 0.0111 | 0.0722 |
| intracellular part | GO:0044424 | C | 3 | 201 | 181 | 0.0143 | 0.0745 |
| ribosome | GO:0005840 | C | 4 | 14 | 24 | 0.0139 | 0.0747 |
| cytosol | GO:0005829 | C | 5 | 19 | 26 | 0.0111 | 0.0748 |
| ribonucleoprotein complex | GO:0030529 | C | 3 | 14 | 24 | 0.0139 | 0.0769 |
| vacuole | GO:0005773 | C | 5 | 4 | 19 | 0.0290 | 0.1435 |
| gene expression | GO:0010467 | P | 4 | 73 | 68 | 0.0288 | 0.1463 |
| transcription, DNA-dependent | GO:0006351 | P | 5 | 53 | 50 | 0.0347 | 0.1590 |
| RNA biosynthetic process | GO:0032774 | P | 5 | 53 | 50 | 0.0347 | 0.1630 |
| RNA metabolic process | GO:0016070 | P | 5 | 53 | 50 | 0.0347 | 0.1672 |
| macromolecule metabolic process | GO:0043170 | P | 3 | 126 | 114 | 0.0460 | 0.2060 |

F, P, and C refer to ‘molecular function’, ‘biological process’, and ‘cellular component’, respectively.
