# Supplementary material for: Comparative Analysis of Transcriptomes in Rhizophoraceae Provides Insights into the Origin and Adaptive Evolution of Mangrove Plants in Intertidal Environments
Source: Front Plant Sci. 2017 May 16;8:795. doi: 10.3389/fpls.2017.00795 (PMC5432612; doi:10.3389/fpls.2017.00795)
Supplement: Supplementary file 2 [file SupplementaryTables7-14.ZIP › Supplementary_Table_S14.docx]

**Supplementary Table S14 | List and annotation of genes under positive selection along the ancestral branch of Rhizophoraceae mangroves.**

| Ortholog ID | NCBI NR annotation | KEGG pathway | | | | | COG annotation |
| --- | --- | --- | --- | --- | --- | --- | --- |
|  |  | Pathway ID | Pathway description | KO ID | KO name | KO description |  |
| GF_7405 | dna polymerase v |  |  |  |  |  |  |
| GF_8253 | chloroplast sensor kinase |  |  |  |  |  | Signal transduction histidine kinase |
| GF_9291 | 50s ribosomal protein l4 | ko03010 | Ribosome | K02926 | RP-L4 | large subunit ribosomal protein L4 | Ribosomal protein L4 |
| GF_9332 | vacuolar atp synthase subunit f | ko00190 | Oxidative phosphorylation | K02151 | ATPeV1F | V-type H+-transporting ATPase subunit F | Archaeal/vacuolar-type H+-ATPase subunit F |
| GF_9460 | eukaryotic translation initiation factor 6 | ko03008 | Ribosome biogenesis in eukaryotes | K03264 | EIF6 | translation initiation factor 6 | Translation initiation factor 6 (eIF-6) |
| GF_9529 | protein |  |  |  |  |  | Uncharacterized integral membrane protein |
| GF_9611 | glutaredoxin |  |  |  |  |  | Glutaredoxin and related proteins |
| GF_9846 | glucosamine 6-phosphate n-acetyltransferase | ko00520 | Amino sugar and nucleotide sugar metabolism | K00621 | GNPNAT1, GNA1 | glucosamine-phosphate N-acetyltransferase | Histone acetyltransferase HPA2 and related acetyltransferases |
| GF_9878 | ubiquitin carboxyl-terminal hydrolase-like protein |  |  |  |  |  |  |
| GF_10047 | uncharacterized protein |  |  |  |  |  |  |
